# Setting priorities for knowledge translation of Cochrane reviews for health equity: Evidence for Equity

**DOI:** 10.1186/s12939-017-0697-5

**Published:** 2017-12-02

**Authors:** Peter Tugwell, Jennifer Petkovic, Vivian Welch, Jennifer Vincent, Zulfiqar A. Bhutta, Rachel Churchill, Don deSavigny, Lawrence Mbuagbaw, Tomas Pantoja

**Affiliations:** 1 Department of Medicine, Faculty of Medicine, University of Ottawa, Ottawa, Canada; 2 Clinical Epidemiology Program, Ottawa Hospital Research Institute, Ottawa, Canada; 3 WHO Collaborating Centre for Knowledge Translation and Health Technology Assessment in Health Equity, Bruyère Research Institute, Ottawa, Canada; 4 Bruyère Research Institute, University of Ottawa, Ottawa, Canada; 5 Center of Excellence in Women and Child Health, The Aga Khan University, Karachi, Pakistan; 6 Centre for Reviews and Dissemination, University of York, York, UK; 7 Department of Epidemiology and Public Health, Swiss Tropical and Public Health Institute, Basel, Switzerland; 8 Department of Health Research Methods, Evidence and Impact, McMaster University, Hamilton, Canada; 9 Department of Family Medicine, Faculty of Medicine, Pontificia Universidad Católica de Chile, Centro Medico San Joaquin, Santiago, Chile

**Keywords:** Equity, Systematic reviews, Priority setting

## Abstract

**Background:**

A focus on equity in health can be seen in many global development goals and reports, research and international declarations. With the development of a relevant framework and methods, the Campbell and Cochrane Equity Methods Group has encouraged the application of an ‘equity lens’ to systematic reviews, and many organizations publish reviews intended to address health equity.

The purpose of the Evidence for Equity (E4E) project was to conduct a priority-setting exercise and apply an equity lens by developing a knowledge translation product comprising summaries of systematic reviews from the Cochrane Library. E4E translates evidence from systematic reviews into ‘friendly front end’ summaries for policy makers.

**Methods:**

The following topic areas with high burdens of disease globally, were selected for the pilot: diabetes/obesity, HIV/AIDS, malaria, nutrition, and mental health/depression. For each topic area, a “stakeholder panel” was assembled that included policymakers and researchers. A systematic search of Cochrane reviews was conducted for each area to identify equity-relevant interventions with a meaningful impact. Panel chairs developed a rating sheet which was used by all panels to rank the importance of these interventions by: 1) Ease of Implementation; 2) Health System Requirements; 3)Universality/Generalizability/Share of Burden; and 4) Impact on Inequities/Effect on equity.

The ratings of panel members were averaged for each intervention and criterion, and interventions were ordered according to the average overall ratings.

**Results:**

Stakeholder panels identified the top 10 interventions from their respective topic areas. The evidence on these interventions is being summarized with an equity focus and the results posted online, at http://methods.cochrane.org/equity/e4e-series.

**Conclusions:**

This method provides an explicit approach to setting priorities by systematic review groups and funders for providing decision makers with evidence for the most important equity-relevant interventions.

**Electronic supplementary material:**

The online version of this article (10.1186/s12939-017-0697-5) contains supplementary material, which is available to authorized users.

## Background

The number of reports of systematic reviews of research has increased from about 80 a year in the late 1980s to more than 8000 a year today [1]. This makes it very difficult for decision makers to keep abreast of the latest evidence. The Campbell and Cochrane Equity Group is committed to finding ways of helping decision makers access and use the evidence on interventions that has impact on health inequities. Health inequities are avoidable differences in health outcomes [2]. The importance of equity in health, wellbeing and wealth is increasingly accepted globally, and it underpins research, global development goals and reports, and international declarations [3–9]. The Campbell and Cochrane Collaborations, and other groups, such as the Alliance for Health Policy and Systems Research and the International Initiative for Impact Evaluation (3ie), publish systematic reviews of the evidence for what works and what does not. There has been an increased emphasis on health equity in systematic reviews with the establishment of a Campbell and Cochrane Equity Methods Group (Equity Methods Group), whose members have provided a framework [10] and methods [11, 12] for applying an ‘equity lens’ to systematic reviews.

However, there is an ongoing need for dissemination and integrated knowledge translation of systematic reviews, to make users aware of knowledge and facilitate its use to improve health and health systems [13–17]. A number of initiatives are currently addressing this challenge, such as the following:

- Evidence Aid review summaries for major healthcare emergencies, including disasters (www.evidenceaid.org/) [18];
- Supporting Policy-relevant Reviews and Trials (SUPPORT) evidence summaries of health systems interventions in low- and middle-income countries, which are based on a simplified version of the Cochrane Summary of Findings Tables (www.supportsummaries.org/);
- Evidence summaries developed by the International Initiative for Impact Evaluation (3ie) in the areas of health, nutrition and population which emphasize photographs and text and are exploring the use of expert commentaries (http://www.3ieimpact.org/en/inform-policy/health-nutrition-and-population/);
- Syntheses of research evidence about governance, financial and delivery arrangements within health systems, and about implementation strategies that can support change in health systems (www.healthsystemsevidence.org/); and
- Countdown to 2030 produces thematic or country-specific briefing notes for policymakers on topics related to maternal, newborn, and child survival (http://countdown2030.org/reports-and-articles/briefing-notes);
  These websites and databases include varying amounts of information related to health equity, such as ‘what works’ for disadvantaged individuals and groups. We developed this Evidence for Equity (E4E) project to focus specifically on equity-relevant interventions. E4E applies an equity lens to systematic reviews through a knowledge translation product comprising summaries of systematic reviews from the Cochrane and Campbell libraries. E4E translates evidence from systematic reviews into “friendly front-end” summaries for policy makers. Building on these other collections of summaries, E4E aims to summarize evidence on interventions that may reduce inequities. The aim of this special collection of systematic review summaries is to provide policy makers, clinicians, and other practitioners, particularly those working in resource-limited settings, with easily accessible, high quality evidence on relevant interventions.

Despite the increased recognition of the importance of knowledge translation of systematic reviews which summarize the totality of the evidence, there is very little done to prioritize topics for focused knowledge translation efforts. The objective of this study was to identify which systematic reviews were highest priority for knowledge translation, with a focus on promoting health equity, in collaboration with policymakers and program managers.

**Table:** 

Take Home Messages
1. For policy makers and program managers in high- or low-/middle-income countries who want to make evidence-based decisions on equity-focused interventions, it is challenging to find evidence on interventions that are effective. 2. This pilot project assessed priority setting methods to identify priority interventions from Cochrane systematic reviews for which there is evidence of a benefit in five topic areas: diabetes/obesity, HIV/AIDS, malaria, nutrition, and depression. 3. This paper presents criteria for priority setting for systematic review groups and funders which may help identify the most important equity-relevant interventions.

## Methods

A steering group of individuals with extensive experience with systematic reviews and knowledge translation methods met face-to-face in London, England in February of 2012. During a two-day meeting, the group decided to focus on a combination of priorities using the Millennium Development Goals as a starting point and expanding on these to also include non-communicable diseases. This resulted in the selection of the following pilot topic areas, each of which has a high burden of disease globally, as indicated by associated disability-adjusted life years (DALYs):

- Diabetes/obesity: For diabetes mellitus, over 59 million DALYs (2.2% total DALYs) as of 2012^16^
- HIV/AIDS: Almost 92 million DALYs (3.4% total DALYs) as of 2012^16^
- Malaria: Over 55 million DALYs (2.0% total DALYs) as of 2012^16^
- Nutrition: For children under 5 years of age, maternal and child undernutrition is responsible for 11% of global DALYs as of 2012 [19]
- Mental health/depression: For unipolar depressive disorders, 76.5 million DALYs (2.8% total DALYs) as of 2012 [20]

In 2015, the United Nations created the global Sustainable Development Goals (SDGs); a group of 17 goals to be met by 2030 [21]. The topic areas listed above are still relevant to the SDGs. Goal number 3 addresses all health priorities and includes reproductive, maternal and child health; communicable and non-communicable diseases; as well as access for all to safe, effective, and affordable medicines and vaccines [21]. In addition, goal number 10 is to reduce inequalities within and between countries and focuses on eliminating inequities based on age, sex, disability, race, ethnicity, origin, religion, and socioeconomic or other status.

Systematic reviews on these five topic areas were retrieved through a search in the Cochrane Library (via Wiley (http://www.cochranelibrary.com/), up to 2013, Issue 6) using relevant key words in the title field and limiting the start date to 2008. The exact search strategies are reported in Additional file 1.

Two independent screeners reviewed the results section (Data and Analysis) of the Cochrane reviews to identify: a) any statistically significant difference in mortality; b) for any other categorical morbidity outcomes besides mortality, Odds Ratio (OR) or Relative Risk (RR) greater than 2 or less than 0.5; [22] c) all statistically significant continuous morbidity outcomes (SMD, MD) that when transformed into ORs were greater than 2. Surrogate outcomes and non-statistically significant effects were excluded. Details of the population, intervention, comparisons, outcomes, and effect size were extracted.

Negative effect sizes that demonstrated benefit were converted to a positive value, by reversing the scale for continuous outcomes or by taking the inverse of dichotomous outcomes (e.g. >1). All effect sizes were converted to odds ratios to allow for comparison across reviews using the formulae provided in the Cochrane Handbook [23]. The results are described as the “converted effect size and confidence interval”.

Five “Stakeholder Panels” were assembled to participate in the priority-setting exercise, each addressing one of the five condition-related topic areas listed above. For each panel, a chair(s) was recruited based on their expertise in one or both conditions and in conducting systematic reviews. The chair(s) helped identify and approach five other policy makers and researchers (stakeholders) to join the panel. Members of these panels were purposefully selected to ensure a variety of policymakers (e.g. national, regional, civil society, NGO) from both HIC and LMIC, with responsibility in the topic area of their panel and with interest in evidence-based policy making.

Stakeholder panel chairs reviewed the initial list of potential interventions and outcomes and eliminated: a) those which are no longer used b) those which could not be implemented globally due to prohibitive costs, especially in resource-constrained settings c) interventions whose outcomes were not meaningfully important.

Chairs collaborated on the development of a rating sheet which was used by other panel members to rank the interventions on a scale from 0 to 4, with 4 denoting an optimal intervention, for four criteria. These criteria were developed based on the Child Health and Nutrition Research Initiative (CHNRI) priority setting exercise [24].

A. Ease of Implementation: Ease with which the intervention can be implemented. Consider whether there is sufficient capacity to implement the intervention.
B. Health System Requirements: Potential effect on the health system. Consider the level of difficulty with intervention delivery, the infrastructure required (human resources, facilities, etc.). Consider the resources available and whether the intervention is affordable.
C. Universality/Generalizability/Share of Burden: Relevance of the intervention to other settings. Is the intervention relevant to most countries? Consider whether the intervention poses safety concerns and whether these may be different in different settings. Rank lower for a less generalizable intervention, or one that applies only to a specific population.
D. Impact on Inequities/Effect on equity: Does the distribution of the disease burden mainly affect the disadvantaged? Are the disadvantaged most likely to benefit from the intervention? Will the intervention improve equity in disease burden distribution long-term? Rank lower for interventions that may increase inequities.

Stakeholder panel members were also asked to note any safety concerns. Finally, they were asked to give an overall rating for each intervention (from 1 to 4 where 1 was the least important intervention and 4 was the most important intervention). Instructions given to stakeholder panel members are provided in Additional file 2.

Lastly, the ratings of all panel members were averaged for each intervention and criteria. We converted the average rating into a score out of 100 for ease of interpretation. This step is different from the CHNRI method which calculates the scores divided by the number of received answers to obtain a percentage of agreement [24]. We ordered interventions according to the average overall rating. We provided these rank-ordered lists to all panel members.

## Results

Each stakeholder panel consisted of at least six members, including the panel chair plus five or more additional experts. The characteristics of our stakeholders are listed in Table 1.

**Table 1:** Characteristics of Participants in all Stakeholder Panels

	N (%)
Total	32
Male	26 (81.25)
Female	6 (18.75)
Country	
High-income	21 (65.6)
Low- and middle-income	11 (34.4)
Australia	1 (3.1)
Argentina	1 (3.1)
Cameroon	2 (6.25)
Canada	6 (18.8)
Chile	1 (3.1)
India	1 (3.1)
Italy	1 (3.1)
Kenya	2 (6.25)
Lebanon	1 (3.1)
Pakistan	1 (3.1)
Peru	1 (3.1)
South Africa	3 (9.4)
Switzerland	3 (9.4)
US	5 (15.63)
UK	3 (9.4)
Role	
Clinician	12 (37.5)
Policy	22 (68.8)
Researcher	30 (93.8)

Note: percentages do not add to 100 since stakeholders could have multiple roles

### Eligible systematic reviews, reaching criteria for important effects

We reviewed all systematic reviews in the areas of depression, malaria, nutrition, diabetes/obesity and HIV in the Cochrane Library from 2008 to 2013. Of these, 96 reviews met the criteria for being relevant to current practice, having an odds ratio > 2 for morbidity, and/or for having a meaningful impact on mortality.

### Consensus ratings

Stakeholder panel members reported that the wide range of interventions and outcomes made ranking difficult and in some cases reported that they gave more priority to interventions with which they were more familiar. We needed to provide additional information for some panel members to complete their rankings. Panel members also reported having some difficulty judging the intervention for some of the criteria without having a particular context or without more details about the intervention (e.g. frequency, delivery method). Additional judgment was needed where interventions may be provided in combinations that may differ depending on the local context. In such situations, panel members were encouraged to think of the real-life practicalities in one of the countries with a high burden of the condition of interest.

Panel members used the full range of the scale from 1 to 4 for each criterion. We did not find evidence of bimodal distributions in the scores that would suggest disagreement within the panel ratings. Furthermore, panel members reached consensus on the top 10 interventions in each panel easily.

### Top-ranked interventions for knowledge translation

Table 2 shows the prioritisation results for diabetes/obesity. See Additional file 3 for the same tables for the other 4 conditions. These show the ratings by the panels on the degree that these systematic reviews merited focus for knowledge translation based on their importance for improving the health of the disadvantaged, based on the four criteria of health system effects, generalizability, impact on health equity and ease of implementation.

**Table 2:** Diabetes/obesity top 10 interventions

	Intervention	Outcome	Feasibility^a^	Deliverability^b^	Universality^c^	Effect on Equity^d^	Overall Rating (%)
1	Sulphonylureas versus insulin	All-cause mortality; best-worst case scenario	Range: 2–4 Total: 75 Rank: 3	Range: 2–4 Total: 75 Rank: 1	Range: 2–4 Total: 75 Rank: 5	Range: 2–4 Total: 70.83 Rank:7	76.67 Rank: 1
2	metformin vs sulphonylureas or insulin.	all cause mortality	Range: 2–4 Total: 87.5 Rank: 1	Range: 2–4 Total: 66.67 Rank: 5	Range: 2–4 Total: 75 Rank: 5	Range: 2–4 Total: 70.83 Rank: 7	75 Rank: 2
3	ACEi versus placebo/no treatment	All cause mortality	Range: 2–4 Total: 80 Rank: 2	Range: 2–4 Total: 75 Rank:1	Range: 3–4 Total: 85 Rank: 1	Range: 2–3 Total: 70 Rank: 8	71.15 Rank: 3
4	Low salt vs high salt diet	Systolic BP	Range: 1–3 Total: 62.5 Rank: 9	Range: 1–4 Total: 71.88 Rank: 2	Range: 2–3 Total: 59.38 Rank: 14	Range: 2–4 Total: 71.88 Rank: 5	77.5 Rank: 4
5	Exercise vs no exercise	Glycated haemoglobin (%)	Range: 1–3 Total: 62.5 Rank: 9	Range: 2–4 Total: 71.88 Rank: 2	Range: 2–3 Total: 71.88 Rank: 6	Range: 2–3 Total: 68.75 Rank: 9	73 Rank: 5
6	Group-based diabetes education programme versus individual routine treatment	reduction in diabetes medication	Range: 1–4 Total: 53.13 Rank: 15	Range: 2–4 Total: 68.75 Rank: 4	Range: 2–3 Total: 65.63 Rank: 9	Range: 2–4 Total: 81.25 Rank: 1	73 Rank: 5
7	Effects of intensive versus brief education in high risk patient samples	Foot ulcer incidence (1-year follow-up)	Range: 1–3 Total: 50 Rank: 16	Range: 2–4 Total: 65.63 Rank: 6	Range: 2–4 Total: 65.63 Rank: 9	Range: 2–4 Total: 78.13 Rank: 3	71 Rank: 6
8	Tight-moderate versus loose glycaemic control	Pre-eclampsia	Range: 0–3 Total: 46.43 Rank: 18	Range: 0–3 Total: 50 Rank: 19	Range: 0–3 Total: 53.57 Rank: 18	Range: 0–3 Total: 53.57 Rank: 18	70.31 Rank: 7
	Very tight versus tight-moderate glycaemic control	Maternal hospitalisation (days)					
	Tight versus moderate glycaemic control	Maternal hypoglycaemia in first half of pregnancy					
9	low glycaemic index (LGI) vs high glycaemic index	large-for-gestational age	Range: 2–4 Total: 68.75 Rank: 5	Range: 2–4 Total: 75 Rank: 1	Range: 2–4 Total: 78.13 Rank: 4	Range: 2–4 Total: 78.13 Rank: 3	67 Rank: 8
10	ACEi versus placebo/no treatment	Systolic BP	Range: 2–4 Total: 75 Rank: 3	Range: 2–4 Total: 68.75 Rank: 4	Range: 2–4 Total: 75 Rank: 5	Range: 2–3 Total: 68.75 Rank: 9	65.38 Rank: 9

^a^Is there sufficient capacity to implement the intervention? Is it feasible to provide required training to staff? Rankings are 0 to 4. 4 = optimal (easier to implement), 0 = more difficult

^b^Consider the level of difficulty with intervention delivery, the infrastructure required (human resources, facilities, etc.). Consider the resources available and whether the intervention is affordable. Rank 0–4, 4 = optimal (easier/fewer health system effects), 0 = more difficult/greater health system effects,

^c^Is the intervention relevant to most countries? Rankings are 0 to 4. 4 = Optimal (more generalizable/population-based, 0 = less generalizable/specific population

^d^Does the distribution of the disease burden affect mainly the disadvantaged? Are the disadvantaged most likely to benefit from the intervention? Will the intervention improve equity in disease burden distribution long-term? Rankings are 0 to 4. 4 = Optimal (more generalizable/population-based, 0 = less generalizable/specific population

## Discussion

With the realisation that single studies, however large, should not drive policy due to the fact that they may not be replicable [25] there has been an exponential increase in systematic reviews. Research community members, especially those working on reducing health inequities, have a responsibility to inform policymakers and their advisors who make decisions on which systematic reviews should be prioritized for knowledge translation for the benefit of the most vulnerable members of their populations. Such global exercises need to be sensitive to major regional differences in needs and perceived priorities.

Our approach differs from other priority-setting exercises because we chose to focus on prioritizing knowledge translation of completed systematic reviews that have the potential to promote health equity. We also involved those who need and use this evidence with researchers and publishers in order to meet the information needs for those making decisions related to equity. The intent is to provide an international platform to deliver summaries from systematic reviews on interventions that impact on health in disadvantaged populations. The target audience includes policymakers, clinicians, regulators, and the general public. This E4E initiative addresses the criticism that Cochrane reviews fail to draw useful conclusions [26] and instead call for more research by prioritizing reviews with potential for health equity impact for knowledge translation and broad dissemination.

Consensus was successfully achieved in identifying the top group of equity-relevant interventions in each of the five pilot areas. The intent was not to focus on specific ranking; rather, it was to provide a matrix across these five criteria to highlight the importance of health equity in decisions on identifying priority interventions given limited resources. The next step is to meet with the relevant Campbell and Cochrane review groups, and other interested systematic review groups, and explore with them whether and how this process can be incorporated into their own priority-setting processes for knowledge translation, as the Cochrane and Campbell Collaborations are both currently developing knowledge translation strategies for their reviews.

Many Cochrane systematic reviews are focused on intervention efficacy, and equity concerns are often more related to intervention implementation and delivery. Therefore, the evidence in the review may not relate to its actual importance in practice. To address this issue, we asked our stakeholders to consider the feasibility of the intervention, deliverability, universality, and effects on health equity. We did not include non-experimental data on harms in this exercise but will include this information in future updates, when available.

Our methods for this priority-setting exercise are similar to those used by other groups, such as Child Health and Nutrition Research Initiative CHNRI [24] and the James Lind Alliance, which uses priority-setting partnerships to develop priorities for ten intervention uncertainties for consideration by research funders [27]. Our approach also aligns with guidance provided by Lavis et al. for health decision makers (policy and programs) which includes using explicit criteria based on the underlying problem and burden of disease and intervention options [28]. Other papers similarly describe priority-setting exercises for research. These methods include surveys and face-to-face consultations and evidence mapping [29].

Informing decision makers should involve providing an easily understood ‘Friendly Front-End’ [13]. Firstly, this derivative summary must provide information on not only the relative effect or statistical significance alone, but also the absolute magnitude of the benefits as well as potential harms, where relevant. Secondly, for those interventions with meaningful, substantive benefit, policymakers also require guidance on: a) ease of implementation of the intervention, including the available capacity and human resources; b) health system requirements and effects on the health system c) universality – i.e., the magnitude of the burden of illness in the country of interest. Finally, policymakers should be informed about whether the intervention will reduce health inequities. There is very little research available on the types of policy summaries and their impact on policy-makers knowledge and decision-making [30].

### Strengths

Each topic area was co-led by an internationally-recognized “content leader” in the respective content field (i.e., depression, diabetes/obesity, HIV, malaria, and nutrition). Each content leader was teamed up with a Cochrane methodologist with expertise in performing systematic reviews in the same area. Each team was composed of a mixture of researchers and policymakers. The explicit focus on equity was helped by the delineation of the three additional criteria: a) the ease of implementation of the intervention, including the available capacity; b) Health system requirements and effects on the health system c) Universality, or the magnitude of the burden of illness in the country of interest. Consensus was achieved remarkably easily on the assessment criteria. Also, disaggregation of the components contributing to the total score did not show any one of the components driving the total score. This may well be different for a specific country or program where there are political factors and competing programs.

### Challenges/weaknesses

It was challenging building the teams as both leaders and team members are in great demand; they are all very busy and typically do not attend Cochrane or other systematic review meetings. There was no financial payment nor academic reward beyond this publication. We initially planned to hold teleconferences but the logistics proved daunting so although we did meet in person or electronically with the leaders, the completion of the worksheets was done asynchronously with the understanding that if there were major disagreements we would set up a teleconference to resolve; however, these were not needed. We had some difficulty getting agreement on the criteria and definitions from our stakeholder panel chairs. As mentioned above, some Stakeholder panel members reported that the wide range of interventions and outcomes made ranking difficult. If the Stakeholder Panels had included different stakeholders this could have changed the priority ranking for some stakeholders. However, since our Panels included diverse individuals and were based on consensus, we feel that the priority lists would have remained similar. Another limitation of our exercise is that we were mostly limited to Cochrane reviews, although the nutrition exercise included some non-Cochrane systematic reviews because the nutrition stakeholder panel chair identified these as interventions with important effects. The other topic areas used only Cochrane systematic reviews. Had additional reviews been included, the results of the exercise may have differed. However, for this exercise we aimed to conduct a priority setting exercise for interventions available in the Cochrane Library.

This paper has focused on the priority setting methods for which the process began in 2013. Since the Cochrane Handbook has not been updated since then, we believe the methods described in this paper would be applicable to the current and future priority setting processes.

### Next steps

Our literature will be updated annually and our E4E summaries will be linked to policy briefs provided through Health Systems Evidence (www.healthsystemsevidence.org), a database containing syntheses of research evidence about governance, financial and delivery arrangements within health systems, and implementation strategies. The summaries will also be linked to the International Initiative for Impact Evaluations (3ie) Briefs (http://www.3ieimpact.org/en/evidence/briefs/), which answer policy questions using impact evaluation results.

This priority-setting exercise will be used to identify Cochrane systematic reviews that will be summarized and added to the E4E Special Collection (http://methods.cochrane.org/equity/e4e-series) (Additional file 4: Screenshot of E4E Landing Page and Additional file 5: Screenshot of HIV Topic Landing Page). To date, there are 25 pilot summaries available on the test website. We plan to conduct user testing of the pilot summaries and will revise the summaries based on the results, then develop new summaries for each of the top 10 interventions identified through the priority-setting exercise.

## Conclusions

This method provides an explicit approach to setting priorities by systematic review groups and funders for providing decision makers with evidence for the most important equity-relevant interventions. Sustainability of this E4E special collection will require partnering with Cochrane and Campbell review groups to continuously identify systematic reviews with potential for important impact on health equity. This could be implemented as part of knowledge translation strategies for these organizations. A first step might be to start with interested review groups, and particularly those covering topics that represent a high burden of disease in low and middle income countries.

## Additional files

Additional file 1: Search Strategies Cochrane Library via Wiley; up to 2013 Issue 6. (DOCX 13 kb)

Additional file 2: Instructions for Priority Setting Exercise. (DOCX 15 kb)

Additional file 3: Priority Setting exercises - Results. (DOCX 115 kb)

Additional file 4: Screenshot of E4E Landing Page and. (PNG 23 kb)

Additional file 5: Screenshot of HIV Topic Landing Page. (PNG 32 kb)

## Abbreviations

CHNRI: Child Health and Nutrition Research Initiative
DALYs: Disability-adjusted life years
E4E: Evidence for Equity
HIC: High income countries
LMIC: Low- and middle-income countries
OR: Odds ratio
RR: Relative risk
