# Supplementary material for: Setting priorities for knowledge translation of Cochrane reviews for health equity: Evidence for Equity
Source: Int J Equity Health. 2017 Dec 2;16:208. doi: 10.1186/s12939-017-0697-5 (PMC5712153; doi:10.1186/s12939-017-0697-5)
Supplement: Supplementary file 1 — Search Strategies Cochrane Library via Wiley; up to 2013 Issue 6. (DOCX 13 kb) [file 12939_2017_697_MOESM1_ESM.docx]

**Additional file 1 – Search Strategies Cochrane Library via Wiley; up to 2013 Issue 6**

Search Name: HIV - E4E manuscript

Last Saved: 29/06/2013 20:39:01.402

Description:

ID Search

#1 HIV:ti (Word variations have been searched)

#2 AIDS:ti

#3 Human immunodeficiency virus:ti

#4 acquired immunodeficiency syndrome:ti

#5 #1 or #2 or #3 or #4 from 2008 to 2013

Search Name: E4E Malaria

Last Saved: 04/07/2013 17:42:31.561

Description: Cochrane Library

ID Search

#1 malaria:ti from 2008 to 2013 (Word variations have been searched)

Search Name: obesity in title from 2008 to 2013 (Word variations have been searched)

Last Saved: 04/07/2013 18:25:23.341

ID Search

#1 obesity:ti from 2008 to 2013 (Word variations have been searched)

Search Name: zinc in title from 2008 to 2013 (Word variations have been searched)

Last Saved: 04/07/2013 18:23:19.139

ID Search

#1 zinc:ti from 2008 to 2013 (Word variations have been searched)

Search Name: vitamin A in title from 2008 to 2013 (Word variations have been searched)

Last Saved: 04/07/2013 18:22:22.839

ID Search

#1 vitamin A:ti from 2008 to 2013 (Word variations have been searched)

Search Name: iodine in title from 2008 to 2013 (Word variations have been searched)

Last Saved: 04/07/2013 18:21:13.889

ID Search

#1 iodine:ti from 2008 to 2013 (Word variations have been searched)

Search Name: depression in title from 2008 to 2013 (Word variations have been searched)

Last Saved: 04/07/2013 18:19:21.573

ID Search

#1 depression:ti from 2008 to 2013 (Word variations have been searched)
