# Supplementary material for: Setting priorities for knowledge translation of Cochrane reviews for health equity: Evidence for Equity
Source: Int J Equity Health. 2017 Dec 2;16:208. doi: 10.1186/s12939-017-0697-5 (PMC5712153; doi:10.1186/s12939-017-0697-5)
Supplement: Supplementary file 2 — Instructions for Priority Setting Exercise. (DOCX 15 kb) [file 12939_2017_697_MOESM2_ESM.docx]

**Additional file 2**

**Instructions for Priority Setting Exercise**

Thank you for agreeing to participate in our E4E Stakeholder Panel for depression. We ask that you complete this priority setting exercise (approximately 1 hour of time) in the next 30 days. We will then set up a consensus call with the entire depression stakeholder panel.

E4E is a knowledge translation project that aims to create user-friendly summaries of systematic reviews for policymakers in low- and middle-income countries. We are focused on interventions that can help reduce inequities in five major health areas: HIV/AIDS, malaria, mental health – depression, nutrition, and public health – diabetes/obesity. We have created five pilot summaries in each topic area and have developed a test website. We would like your help identifying which interventions are most important to make accessible to policymakers making decisions about interventions to reduce health equity.

***Documents needed:***
To complete the priority setting exercise you will need the Word Document called: *E4E depression rating sheet*

This file contains 11 columns.

The first three columns include the title of a systematic review, the intervention or interest, and the outcome which has been selected for its clinical importance and the size of its effect.

Effectiveness: This is the effect size listed in the systematic review – all effect sizes have been converted to odds ratios to assist with comparisons. The interventions in the table have been ordered according to effect size.

***Priority Setting***

The final five columns in the table are empty so that you can use them to rank the interventions on a scale of 0-4.

Ease of Implementation: This refers to the ease with which the intervention can be implemented. Is there sufficient capacity to implement the intervention? Is it feasible to provide required training to staff? Rank 0 if the intervention would be most/more difficult. Rank 4 for the optimal interventions.

Health System Effects: This refers to the potential effect on the health system. Consider the level of difficulty with intervention delivery, the infrastructure required (human resources, facilities, etc.). Consider the resources available and whether the intervention is affordable. Rank 0 if the intervention would be most/more difficult. Rank 4 for an optimal intervention.

Generalizability/Share of Burden: This refers to the relevance of the intervention to other settings. Is the intervention relevant to most countries? Consider whether the intervention poses safety concerns and whether these may be different in different settings. Rank 0 for a less generalizable intervention (or one that applies only to a specific population). Rank 4 for an optimal intervention.

Impact on Inequities: Does the distribution of the disease burden affect mainly the disadvantaged? Are the disadvantaged most likely to benefit from the intervention? Will the intervention improve equity in disease burden distribution long-term? Rank 0 for interventions that may increase inequities. Rank 4 for optimal interventions that would decrease inequities.

Based on your overall opinion of what would be the most important priority interventions, please provide an overall ranking for each review in the final column. There is a row at the bottom for you to leave overall comments.

Based on your ranking we will start with the 10 interventions that are the most important to be summarized on the E4E website for policy makers in low- and middle-income countries.

When you are finished, please send your completed ranking sheet to Jennifer Petkovic ([jennifer.petkovic@uottawa.ca](mailto:jennifer.petkovic@uottawa.ca))

THANK YOU!
