# Supplementary material for: Setting priorities for knowledge translation of Cochrane reviews for health equity: Evidence for Equity
Source: Int J Equity Health. 2017 Dec 2;16:208. doi: 10.1186/s12939-017-0697-5 (PMC5712153; doi:10.1186/s12939-017-0697-5)
Supplement: Supplementary file 3 — Priority Setting exercises - Results. (DOCX 115 kb) [file 12939_2017_697_MOESM3_ESM.docx]

**Additional file 3: Priority Setting exercises - Results**

**Depression**

|  | Review Title | Intervention | Outcome | Original effect size | Effect Size | Feasibility^[[1]](#footnote-1)^ | Deliverability ^[[2]](#footnote-2)^ | Universality ^[[3]](#footnote-3)^ | Effect on Equity^[[4]](#footnote-4)^ | Overall rating (%) |
| --- | --- | --- | --- | --- | --- | --- | --- | --- | --- | --- |
| 1 | Antidepressants versus placebo for depression in primary care | TCAs versus placebo | Depression symptoms at post-treatment | Original SMD  -0.49 [ -0.67, -0.32 ] | OR 2.43  ***Favours treatment*** | Range: 2-4  Total: 59.38 Rank: 1 | Range: 2-4  Total: 59.38  Rank: 1 | Range: 2-4  Total: 62.5  Rank: 3 | Range: 2-4  Total: 59.38 Rank: 2 | 86  Rank: 1 |
| 2 | Low dosage tricyclic antidepressants for depression | ANY DEPRESSION: Low dosage TCA vs Placebo | Depression improved (per protocol): At three to twelve months | Original RR  2.14 [ 1.41, 3.26 ] | OR 3.64 (1.82-7.29)  ***Favours low dosage*** | Range: 1-4  Total: 50  Rank: 3 | Range: 1-4  Total: 50  Rank: 4 | Range: 1-4  Total: 50  Rank: 7 | Range: 2-3  Total: 46.88  Rank: 5 | 76  Rank: 2 |
| 3 | Psychosocial and psychological interventions for treating postpartum depression | Any interventions versus usual care - all trials | Evidence of depression at final assessment within first year | Original RR  0.70 (0.60-0.81) | OR 2.09 (1.61-2.74) ***favours treatment*** | Range: 1-3  Total: 46.88  Rank: 4 | Range: 2-4  Total: 53.13  Rank: 3 | Range: 2-3  Total: 56.25  Rank: 5 | Range: 3-4  Total: 65.63  Rank: 1 | 73.33  Rank: 3 |
|  |  |  | Evidence of depression at final assessment within first year: Edinburgh Postnatal Depression Scale (EPDS)>12 | Original RR  0.44 [ 0.24, 0.80 ] | OR 3.83 (1.49-9.86)  ***Favours treatment*** |  |  |  |  |  |
| 4 | Antidepressants for depression in physically ill people | SSRI vs placebo | Response to treatment (>18 weeks). SSRI versus placebo |  | OR  2.44 [ 1.31, 4.55 ]  ***Favours SSRI*** | Range: 1-4  Total: 50  Rank: 3 | Range: 1-4  Total: 56.25  Rank: 2 | Range: 1-4  Total: 59.38  Rank: 4 | Range: 1-4  Total: 53.13  Rank: 3 | 72  Rank: 4 |
| 5 | Antidepressants for depression in physically ill people | TCA vs placebo | Response to treatment (4-5 weeks). TCA versus placebo |  | OR  4.79 [ 1.86, 12.37 ]  ***Favours TCA*** | Range: 1-4  Total: 50  Rank: 3 | Range: 1-4  Total: 56.25  Rank: 2 | Range: 1-4  Total: 56.25  Rank: 5 | Range: 1-4  Total: 50  Rank: 4 | 68.67  Rank: 5 |
| 6 | Psychological and educational interventions for preventing depression in children and adolescents | Psychological/educational intervention versus no intervention/wait-list/usual care | Depression scores (by intervention) | Original SMD  -0.21 [ -0.28, -0.15 ] | OR 1.46  ***Favours Psychological/educational intervention*** | Range: 1-3  Total: 31.25  Rank: 9 | Range: 1-4  Total: 43.75  Rank: 6 | Range: 2-4  Total: 56.25  Rank: 5 | Range: 2-4  Total: 59.38  Rank: 2 | 66  Rank: 6 |
|  |  |  | Depression scores (by intervention) at 12 months | Original SMD  -0.10 [ -0.18, -0.02 ] | OR 1.20  ***Favours Psychological/educational intervention*** |  |  |  |  |  |
|  |  |  | Depressive disorder (by intervention) at 36 months | Original SMD  -0.10 [ -0.19, -0.02 ] | OR 1.20  ***Favours Psychological/educational intervention*** |  |  |  |  |  |
| 7 | Collaborative care for depression and anxiety problems | Collaborative care vs usual care | Improvement in depression symptoms: 13 to 24 months | Original SMD  -0.35 [ -0.46, -0.24 ] | OR 1.89  ***Favours collaborative care*** | Range: 1-3  Total: 34.38  Rank: 8 | Range: 1-4  Total: 46.88  Rank: 5 | Range: 2-4  Total: 53.13  Rank: 6 | Range: 2-3  Total: 53.13  Rank: 3 | 58  Rank: 7 |
| 8 | Exercise for depression | Exercise versus ’no treatment’ | Reduction in depression symptoms follow-up | Original SMD  - 0.39 [ -0.69, -0.09 ] | OR 2.03  ***Favours exercise*** | Range: 1-4  Total: 46.88  Rank: 4 | Range: 2-4  Total: 59.38  Rank: 1 | Range: 2-4  Total: 62.5  Rank: 3 | Range:2-4  Total: 53.13  Rank: 3 | 54  Rank: 8 |
| 9 | Relaxation for depression | Relaxation + medication versus medication alone | Depression scores - self rated post intervention (SMD) | Original SMD  -0.90 [ -1.56, -0.24 ] | OR 5. 12  ***Favours relaxation+ medication*** | Range: 2-3  Total: 46.88  Rank: 4 | Range: 2-4  Total: 56.25  Rank: 2 | Range: 2-4  Total: 62.5  Rank: 3 | Range: 2-4  Total: 59.38  Rank: 2 | 54  Rank: 8 |
| 10 | Relaxation for depression | Relaxation versus wait-list, no treatment or minimal treatment | Depression scores - self rated follow-up (short-term) | Original SMD  -0.74 [ -1.34, -0.15 ] | OR 3.83  ***Favours relaxation*** | Range: 1-3  Total: 43.75  Rank: 5 | Range: 1-4  Total: 46.88  Rank: 5 | Range: 3-4  Total: 65.63  Rank: 1 | Range: 2-4  Total: 59.38  Rank: 2 | 54  Rank: 8 |
| 11 | Relaxation for depression | Relaxation versus medication | Depression scores - self rated follow-up (short-term) | SMD  0.50 [ 0.05, 0.95 ] | OR 2.48  ***Favours medication*** | Range: 1-4  Total: 53.13  Rank: 2 | Range: 2-3  Total: 46.88  Rank: 5 | Range: 3-4  Total: 65.63 Rank: 2 | Range: 2-3  Total: 53.13  Rank: 3 | 50.67  Rank: 9 |
| 12 | Pharmacological treatment for psychotic depression | amitriptyline + perphenazine versus perphenazine | Hamilton Rating Scale for Depression (HRSD 17) <7 and no delusions | Original RR  3.61 [ 1.23, 10.56 ] | OR 8.17 (1.79-37.33)  ***Favours combination (amitriptyline +perphenazine)*** | Range: 2-4  Total: 50  Rank: 3 | Range: 2-4  Total: 53.13  Rank: 3 | Range: 1-3  Total:37.5  Rank: 11 | Range: 1-3  Total: 40.63  Rank: 7 | 50  Rank: 10 |
|  |  | imipramine versus mirtazine | Hamilton Rating Scale for Depression (HRSD-17)  <50% | Original RR  3.00 [ 1.01, 8.95 ] | OR 6 (1.17-30.73)  ***Favours imipramine*** |  |  |  |  |  |
| 13 | Antidepressants for depression in physically ill people | Antidepressants vs. placebo | Response to treatment (>18 weeks). Antidepressants versus placebo |  | OR  2.13 [ 1.31, 3.47 ]  ***Favours antidepressants*** | Range: 1-4  Total: 46.88  Rank: 4 | Range: 1-4  Total: 53.13  Rank: 3 | Range: 1-4  Total: 56.25  Rank: 5 | Range: 1-4  Total: 53.13  Rank: 3 | 45.33  Rank: 11 |
| 14 | Relaxation for depression | Relaxation versus psychological treatment | Depression scores - self rated follow-up post intervention | SMD 0.36 (0.07-0.65) | OR 1.99  ***Favours psychological*** | Range: 1-3  Total: 40.63  Rank: 6 | Range: 2-3  Total:43.75  Rank: 6 | Range: 2-4  Total: 56.25  Rank: 5 | Range: 2-3  Total: 50  Rank: 4 | 44  Rank: 12 |
| 15 | Sertraline versus other antidepressive agents for depression | Sertraline vs amitriptyline | Standardised mean difference at endpoint (6 - 12 weeks) reduction of depressive symptoms | SMD  0.18 [ 0.04, 0.32 ] | OR 1.39  ***Favours TCA*** | Range: 1-4  Total: 53.13  Rank: 2 | Range: 1-4  Total: 53.13  Rank: 3 | Range: 1-3  Total:46.88  Rank: 8 | Range: 1-3  Total: 43.75  Rank: 6 | 44  Rank:12 |
| 16 | Sertraline versus other antidepressive agents for depression | Sertraline vs imipramine | Acute phase response (50% reduction Hamilton rating scale depression)(6-12 weeks) |  | OR 0.80 (0.57-1.12) ***(favours sertraline)*** | Range: 1-4  Total: 53.13  Rank: 2 | Range: 1-4  Total: 43.75  Rank: 6 | Range: 1-4  Total: 43.75  Rank: 9 | Range: 1-3  Total: 40.63  Rank: 7 | 41.33  Rank: 13 |
|  |  |  | Side effect - Participants with at least one Treatment ending adverse event | Original OR 0.17 [ 0.09, 0.32 ] | OR  5.88 (3.13-11.11)  ***Favours sertraline*** |  |  |  |  |  |
| 17 | Antidepressants versus placebo for depression in primary care | SSRIs versus placebo | Withdrawal from trials at post-treatment: Adverse effects | Original RR  2.44 [ 1.22, 4.86 ] | OR 2.50 (1.22-5.13) ***favours placebo*** | Range: 0-4  Total: 50  Rank: 3 | Range: 0-4  Total: 53.13  Rank: 3 | Range: 0-4  Total: 53.13  Rank: 6 | Range: 0-4  Total: 43.75  Rank: 6 | 40  Rank: 14 |
| 18 | Tryptophan and 5-Hydroxytryptophan for depression | L-Tryptophan and 5-HTP versus placebo for the treatment of depression | Numbers of responders (treatment of depression) |  | OR 4.10 [ 1.28, 13.15 ]  ***Favours L-Tryptophan and 5-HTP*** | Range: 1-3  Total: 37.5  Rank: 7 | Range: 1-3  Total: 34.38  Rank: 9 | Range: 1-4  Total: 43.75  Rank: 9 | Range: 1-3  Total: 31.25  Rank: 10 | 38.46  Rank: 15 |
| 19 | Antidepressants plus benzodiazepines for major depression | ANTIDEPRESSANT+BENZO Vs ANTIDEPRESSANT ALONE | Depression severity: at 4 weeks | Original SMD -0.29 [-0.51, -0.08] | OR 1.49 ***Favours Antidepressant+benzo*** | Range: 1-4  Total: 46.88  Rank: 4 | Range: 1-4  Total: 43.75  Rank: 6 | Range: 1-4  Total: 43.75 Rank: 9 | Range: 1-3  Total: 37.5  Rank: 8 | 36  Rank: 16 |
|  |  |  | Anxiety severity: at six to eight weeks | Original MD  -0.50 [ -0.94, -0.07 ] | OR 2.04  ***Favours combination*** |  |  |  |  |  |
| 20 | Mirtazapine versus other antidepressive agents for depression | Mirtazapine vs SSRIs | Primary outcome: response (at least 50% reduction in the score on the Hamilton Rating Scale for Depression) at 2 weeks |  | OR 1.57 [1.30, 1.88]  ***Favours mirtazapine*** | Range: 1-4  Total: 34.38  Rank: 8 | Range: 1-3  Total: 37.5  Rank: 8 | Range: 1-4  Total: 40.63  Rank: 10 | Range: 1-3  Total: 31.25  Rank: 10 | 34.62  Rank: 17 |
| 21 | St John’s wort for major depression | Hypericum mono-preparations vs. placebo | Remission (HAMD score < 8 or < 7) |  | OR 2.77 [ 1.80, 4.26 ]  ***Favours St. John’s wort*** | Range: 1-3  Total: 40.63  Rank: 6 | Range: 1-3  Total: 37.5  Rank: 8 | Range: 1-4  Total: 43.75  Rank: 9 | Range: 1-3  Total: 40.63  Rank: 7 | 33.33  Rank: 18 |
|  |  |  | Difference HAMD (Hamilton Rating Scale for Depression) baseline - end of treatment | Original MD  -3.03 [ -4.67, -1.39 ] | OR 1.38  ***Favours St. John’s wort*** |  |  |  |  |  |
|  |  | Hypericum mono-preparations vs. standard antidepressants. | Mean D-S (Depression Scale von Zerssen) scores after therapy | MD  2.66 [ 0.83, 4.50 ] | OR 1.72  ***Favours standard antidepressants*** |  |  |  |  |  |
| 22 | Antidepressants for depression in physically ill people | Mianserin versus placebo | Response to treatment (4-5 weeks). Mianserin versus placebo |  | OR  2.91 [ 1.12, 7.52 ]  ***Favours Mianserin*** | Range: 1-3  Total: 40.63  Rank: 6 | Range: 1-3  Total: 40.63  Rank: 7 | Range: 1-4  Total: 43.75  Rank: 9 | Range: 1-3  Total: 43.75  Rank: 6 | 32  Rank: 19 |
| 24 | Mirtazapine versus other antidepressive agents for depression | Mirtazapine vs Paroxetine | Subgroup analysis: Response at 2 weeks (at least 50% reduction in the score on the Hamilton Rating Scale for Depression): Treatment settings: Outpatients in primary care |  | OR  4.38 [ 1.69, 11.35 ]  ***Favours paroxetine*** | Range: 1-3  Total: 34.38  Rank: 8 | Range: 1-4  Total: 37.5  Rank: 8 | Range: 1-4  Total: 37.5  Rank: 11 | Range: 1-4  Total: 31.25  Rank: 10 | 30.77  Rank: 20 |
| 25 | Escitalopram versus other antidepressive agents for depression | Escitalopram versus Citalopram | reduction of depressive symptoms | Original SMD  -0.17 [ -0.30, -0.04 ] | OR 1.36  ***Favours escitalopram*** | Range: 1-4  Total: 40.63  Rank: 6 | Range: 1-3  Total: 34.38  Rank: 9 | Range: 1-4  Total: 34.38  Rank: 12 | Range: 1-3  Total: 28.13  Rank: 11 | 30.77  Rank: 20 |
| 26 | Escitalopram versus other antidepressive agents for depression | Escitalopram versus Fluoxetine | reduction of depressive symptoms | Original SMD  -0.17 [ -0.32, -0.03 ] | OR 1.36  ***Favours escitalopram*** | Range: 1-4  Total: 40.63 Rank: 6 | Range: 1-4  Total: 34.38  Rank: 9 | Range: 1-3  Total: 34.38  Rank: 12 | Range: 1-4  Total: 31.25  Rank: 10 | 30.77  Rank: 20 |
| 27 | Milnacipran versus other antidepressive agents for depression | Milnacipran vs TCAs | Response at acute phase (6-12 wks) | Original OR 0.87 [0.59, 1.30] | OR 1.15  (0.77-1.69)  ***Favours TCAs*** | Range: 1-3  Total: 31.25  Rank: 9 | Range: 1-3  Total: 28.13  Rank: 11 | Range: 1-4  Total: 31.25  Rank: 13 | Range: 1-3  Total: 28.13  Rank: 11 | 26.92  Rank: 21 |
| 28 | Alprazolam for depression | Alprazolam versus placebo | 50% improvement versus less than 50% improvement RR | Original RR  2.47 [ 1.78, 3.43 ] | OR 4.15 (2.53-6.79)  ***Favours alprazolam*** | Range: 1-3  Total: 31.25  Rank: 9 | Range: 1-3  Total: 31.25  Rank: 10 | Range: 1-4  Total: 31.25  Rank: 13 | Range: 1-2  Total: 28.13  Rank: 11 | 19.23  Rank: 22 |
| 29 | Alprazolam for depression | Alprazolam versus amitriptyline | 50% improvement vs less than 50% improvement RD (subgrouped by TCA comparator) | Original RD  -0.19 [ -0.30, -0.07 ] | OR 1.36 (0.76-2.43)  ***Favours amitriptyline*** | Range: 1-3  Total: 34.38  Rank: 8 | Range: 1-4  Total: 31.25  Rank: 10 | Range: 1-4  Total: 31.25  Rank: 13 | Range: 1-4  Total: 37.5  Rank: 8 | 19.23  Rank: 22 |

**Diabetes/Obesity**

|  | Review Title | Intervention | Outcome | Original effect size | Effect Size | Feasibility^[[5]](#footnote-5)^ | Deliverability ^[[6]](#footnote-6)^ | Universality ^[[7]](#footnote-7)^ | Effect on Equity^[[8]](#footnote-8)^ | Overall Rating (%) |
| --- | --- | --- | --- | --- | --- | --- | --- | --- | --- | --- |
| 1 | Sulphonylurea monotherapy for patients with type 2 diabetes mellitus | Sulphonylureas versus insulin | All-cause mortality; best-worst case scenario | RR 0.13 (0.02-0.95)  ***Favours sulphonylureas*** | OR 9.26 (1.08-79.21)  ***Favours sulphonylureas*** | Range: 2-4 Total: 75  Rank: 3 | Range: 2-4 Total: 75  Rank:1 | Range: 2-4 Total: 75  Rank:5 | Range: 2-4 Total: 70.83  Rank:7 | 76.67  Rank: 1 |
| 2 | Metformin monotherapy for type 2 diabetes mellitus | metformin vs sulphonylureas or insulin. | all cause mortality | RR 0.73 (0.55-0.97)  ***Favours metformin*** | OR 1.46 (1.04-2.05)  ***Favours metformin*** | Range: 2-4 Total: 87.5  Rank: 1 | Range: 2-4 Total: 66.67  Rank:5 | Range: 2-4 Total: 75  Rank:5 | Range: 2-4 Total: 70.83  Rank:7 | 75  Rank: 2 |
| 3 | Antihypertensive agents for preventing diabetic kidney disease | ACEi versus placebo/no treatment | All cause mortality | RR 0.84, 95% CI 0.73 to 0.97 | OR 1.19 (1.02-1.38) | Range: 2-4 Total: 80  Rank: 2 | Range: 2-4 Total:75  Rank:1 | Range: 3-4 Total: 85  Rank:1 | Range: 2-3 Total: 70  Rank:8 | 71.15  Rank: 3 |
| 4 | Altered dietary salt intake for preventing and treating diabetic kidney disease | Net change with altering salt diet (low salt vs high salt) | Systolic BP | MD -7.04 [-8.71, -5.38]  ***Favours low salt*** | OR 2.74  ***Favours low salt*** | Range: 1-3 Total: 62.5  Rank:9 | Range: 1-4 Total: 71.88  Rank:2 | Range: 2-3 Total: 59.38  Rank:14 | Range: 2-4 Total: 71.88  Rank: 5 | 77.5  Rank: 4 |
| 5 | Exercise for type 2 diabetes mellitus | Exercise vs no exercise | Glycated haemoglobin (%) | MD -0.62 [-0.91, -0.33]  ***Favours exercise*** | OR 2.22  ***Favours exercise*** | Range: 1-3 Total: 62.5  Rank:9 | Range: 2-4 Total: 71.88  Rank:2 | Range: 2-3 Total: 71.88  Rank:6 | Range: 2-3 Total: 68.75  Rank:9 | 73  Rank: 5 |
| 6 | Group based training for self-management strategies in people with type 2 diabetes mellitus | Group-based diabetes education programme versus individual routine treatment | reduction in diabetes medication | OR  11.79 (5.17-26.90)  ***Favours group educ*** | OR >10  ***Favours group educ*** | Range: 1-4 Total: 53.13  Rank:15 | Range: 2-4 Total: 68.75  Rank:4 | Range: 2-3 Total: 65.63  Rank:9 | Range: 2-4 Total: 81.25  Rank:1 | 73  Rank: 5 |
| 7 | Patient education for preventing diabetic foot ulceration | Effects of intensive versus brief education in high risk patient samples | Foot ulcer incidence (1-year follow-up) | RR 0.31 [0.14, 0.66]  ***favours intensive*** | OR 3.64 (1.60-8.28)  ***favours intensive*** | Range: 1-3 Total: 50  Rank:16 | Range: 2-4 Total: 65.63  Rank:6 | Range: 2-4 Total: 65.63  Rank:9 | Range: 2-4 Total: 78.13  Rank:3 | 71  Rank: 6 |
| 8 | Different intensities of glycaemic control for pregnant women with pre-existing diabetes | Tight-moderate versus loose glycaemic control | Pre-eclampsia | RR 0.11 (0.01, 0.99)  ***Favours tight-mod*** | OR 11 (1.05-115.51)  ***Favours tight-mod*** | Range: 0-3 Total: 46.43  Rank:18 | Range: 0-3 Total: 50  Rank:19 | Range: 0-3 Total: 53.57  Rank:18 | Range: 0-3 Total: 53.57  Rank:18 | 70.31  Rank: 7 |
|  |  | Very tight versus tight-moderate glycaemic control | Maternal hospitalisation (days) | MD  10.60 (9.53, 11.67)  ***Favours tight-mod*** | OR 5.99  ***Favours tight-mod*** |  |  |  |  |  |
|  |  | Tight versus moderate glycaemic control | Maternal hypoglycaemia in first half of pregnancy | RR 26.47 (1.61, 435.38)  ***Favours mod*** | OR >10  (1.47-565.29)  ***Favours mod*** |  |  |  |  |  |
| 9 | Dietary advice in pregnancy for preventing gestational diabetes mellitus | low glycaemic index (LGI) vs high glycaemic index | large-for-gestational age | RR 0.09 (0.01-0.69)  ***Favours LGI*** | OR 15.5 (1.84-130.58)  ***Favours LGI*** | Range: 2-4 Total: 68.75  Rank:5 | Range: 2-4 Total: 75  Rank:1 | Range: 2-4 Total: 78.13  Rank:4 | Range: 2-4 Total: 78.13  Rank:3 | 67  Rank: 8 |
| 10 | Antihypertensive agents for preventing diabetic kidney disease | ACEi versus placebo/no treatment | Systolic BP | SMD 5.37 [7.12, 3.62]  ***Favours ACEi*** | OR >10  ***Favours ACEi*** | Range: 2-4 Total: 75  Rank:3 | Range: 2-4 Total: 68.75  Rank:4 | Range: 2-4 Total: 75  Rank:5 | Range: 2-3 Total: 68.75  Rank:9 | 65.38  Rank: 9 |
| 11 | Antihypertensive agents for preventing diabetic kidney disease | ACEi versus CCB | Systolic BP | SMD 4.0 [1.59, 6.41]  ***Favours CCB*** | OR >10  ***Favours CCB*** | Range: 2-4 Total: 80  Rank:2 | Range: 2-4 Total: 70  Rank:3 | Range: 2-4 Total: 80  Rank:3 | Range: 2-3 Total: 65  Rank:11 | 65.38  Rank: 9 |
| 12 | Alpha-glucosidase inhibitors for type 2 diabetes mellitus | Acarbose vs. nateglinide/repaglinide | Change in body weight | MD  -0.68 (-1.30, -0.06)  ***Favours acarbose*** | OR 1.82  ***Favours acarbose*** | Range: 0-3 Total: 54.17  Rank:13 | Range: 0-4 Total: 58.33  Rank:13 | Range: 0-4 Total: 58.33  Rank:15 | Range: 0-4 Total: 54.17  Rank:17 | 65  Rank: 10 |
| 13 | Colesevelam for type 2 diabetes mellitus | Colesevelam versus placebo | Mean change in fasting blood glucose from baseline to endpoint | MD -14.66 (-21.47, -7.84)  ***Favours Colesevelam*** | OR 1.69  ***Favours Colesevelam*** | Range: 0-4 Total: 65  Rank:7 | Range: 0-4 Total: 55  Rank:16 | Range: 0-4 Total: 70  Rank:8 | Range: 0-4 Total: 60  Rank:13 | 63.33  Rank: 11 |
| 14 | Exercise or exercise and diet for preventing type 2 diabetes mellitus | Exercise+diet vs standard recommendations | Diabetes incidence | OR 0.51 (0.40-0.65) ***favours treatment*** | OR 0.51 (0.40-0.65) ***favours treatment*** | Range: 1-3 Total: 56.25  Rank:12 | Range: 1-3 Total: 56.25  Rank:15 | Range: 1-4 Total: 71.88  Rank:6 | Range: 3 Total: 75  Rank:4 | 63  Rank: 12 |
| 15 | Interventions for treating obesity in children | Lifestyle interventions in children 12 years and older  (behavioural group intervention vs self-help control) | Change in BMI at twelve months follow up | MD -3.27 [ -3.38, -3.17 ]  ***Favours group intervention*** | OR>10  ***Favours group intervention*** | Range: 2-3 Total: 53.13  Rank:13 | Range: 1-3 Total: 53.13  Rank:18 | Range: 2-4 Total: 65.63  Rank:9 | Range: 2-4 Total: 71.88  Rank:5 | 63  Rank: 12 |
| 16 | Interventions for improving adherence to treatment recommendations in people with type 2 diabetes mellitus | education/facilitation | HbA1c | MD -0.36 (-0.57, -0.15)  ***Favours intervention*** | OR 1.40  ***Favours intervention*** | Range: 1-3 Total: 62.5  Rank:9 | Range: 2-4 Total: 62.5  Rank:10 | Range: 2-3 Total: 65.63  Rank:9 | Range: 2-4 Total: 75  Rank:4 | 62  Rank: 13 |
| 17 | Sulphonylurea monotherapy for patients with type 2 diabetes mellitus | Second-generation sulphonylureas versus first-generation sulphonylureas | All cause mortality | RR 0.90 [0.72, 1.11]  ***Favours 2^nd^ generation*** | OR 1.15 (0.87-1.51)  ***Favours 2^nd^ generation*** | Range: 0-4 Total: 75  Rank:3 | Range: 2-4 Total: 62.5  Rank:10 | Range: 2-4 Total: 70.83  Rank:7 | Range: 0-4 Total: 62.5  Rank:12 | 60  Rank: 14 |
|  |  |  | Change in fasting blood glucose from baseline (mmol/L) | MD 0.62 (0.31-0.94)  ***Favours 1^st^ generation*** | OR 1.58  ***Favours 1^st^ generation*** |  |  |  |  |  |
| 19 | Computer-based diabetes self-management interventions for adults with type 2 diabetes mellitus | Computer intervention vs control | HbA1c | MD -0.21 (-0.37, -0.05)  ***Favours intervention*** | OR 1.67 ***Favours intervention*** | Range: 1-4 Total: 53.13  Rank:15 | Range: 0-4 Total: 50  Rank:19 | Range: 2-3 Total: 56.25  Rank:17 | Range: 1-3 Total: 56.25  Rank:16 | 58  Rank: 15 |
| 20 | Exercise or exercise and diet for preventing type 2 diabetes mellitus | Exercise+diet vs standard recommendations | Mean differences between groups in waist circumference (cm) | MD -3.90 (-5.90, -1.91)  ***Favours intervention*** | OR 1.29  ***Favours intervention*** | Range: 1-3 Total: 53.57  Rank:14 | Range: 2-3 Total: 57.14  Rank:14 | Range: 1-3 Total: 64.29  Rank:10 | Range: 2-3 Total: 71.43  Rank:6 | 55.88  Rank: 16 |
| 21 | Screening and subsequent management for gestational diabetes for improving maternal and infant health | Risk factor versus universal screening | Diagnosis of gestational diabetes | RR 0.44 (0.26 -0.75)  ***Favours universal*** | OR 2.30 (1.35-3.95)  ***Favours universal*** | Range: 1-4 Total: 71.88  Rank:4 | Range: 1-4 Total: 65.63  Rank:6 | Range: 2-4 Total: 75  Rank:5 | Range: 1-4 Total: 71.88  Rank:5 | 54  Rank: 17 |
| 22 | Antihypertensive agents for preventing diabetic kidney disease | ARB versus placebo/no treatment | Systolic BP | SMD -3.28 [-5.29, -1.27]  ***Favours ARB*** | OR >10  ***Favours ARB*** | Range: 2-4 Total: 80  Rank:2 | Range: 2-4 Total:65  Rank:7 | Range: 2-4 Total: 80  Rank:3 | Range: 2-3 Total:65  Rank:11 | 53.85  Rank: 18 |
| 23 | Individual patient education for people with type 2 diabetes mellitus | individual diabetes care vs group education | HbA1c (6-9 months) (r=0.75) | MD  0.81 (0.34-1.29)  ***Favours indiv. educ*** | OR 2.70  ***Favours indiv. educ*** | Range: 2-3 Total:60.71  Rank:10 | Range: 2-3 Total: 60.71  Rank:11 | Range: 2-3 Total: 64.29  Rank:10 | Range: 2-4 Total: 78.57  Rank:2 | 53.75  Rank: 19 |
| 24 | Dietary advice for treatment of type 2 diabetes mellitus in adults | dietary advice vs dietary advice + exercise | weight at 12 mos. | MD -6.74 (-11.72, -1.76)  ***Favours dietary advice + exercise*** | OR 2.30  ***Favours dietary advice + exercise*** | Range: 0-4 Total: 53.13  Rank:15 | Range: 0-4 Total: 62.5  Rank:10 | Range: 0-4 Total:59.38  Rank:14 | Range: 0-4 Total: 59.38  Rank:15 | 53.75  Rank: 20 |
| 25 | Different strategies for diagnosing gestational diabetes to improve maternal and infant health | 75 g oral GTT v 100 g oral GTT | Diagnosis of gestational diabetes | RR 2.55 [0.96, 6.75] | OR 2.75 (0.98-7.77) | Range: 2-4 Total: 67.86  Rank:6 | Range: 1-3 Total: 64.23  Rank:9 | Range: 1-3 Total: 60.71  Rank:12 | Range: 1-3 Total: 67.86  Rank:10 | 53.75  Rank: 20 |
| 26 | Low glycaemic index, or low glycaemic load, diets for diabetes mellitus | Low glycaemic index or low glycaemic load diet versus other diet | Glycated haemoglobin (%HbA1c) | MD -0.50 (-0.81, -0.20)  ***Favours low glycaemic*** | OR 2.12  ***Favours low glycaemic*** | Range: 1-3 Total: 40.63  Rank:20 | Range: 1-4 Total: 59.38  Rank:12 | Range: 2-3 Total: 62.5  Rank:11 | Range: 2-3 Total: 62.5  Rank:12 | 52  Rank: 21 |
| 27 | Chinese herbal medicines for type 2 diabetes mellitus | Herbal medicine versus placebo | Normalisation of fasting blood glucose (< 7.2 mmol/L) | RR 2.03 [1.39, 2.98]  ***Favours herbs*** | OR 2.43 (1.51-3.92)  ***Favours herbs*** | Range: 1-3 Total: 53.13  Rank:15 | Range: 1-4 Total: 59.38  Rank:12 | Range: 1-3 Total: 53.13  Rank:19 | Range: 1-3 Total: 62.5  Rank:12 | 52  Rank: 21 |
| 28 | Computer-based diabetes self-management interventions for adults with type 2 diabetes mellitus | Computer intervention vs control | pooled effect on diet (estimated daily fat intake + change in weekly calorie intake) | MD -0.29 (-0.43--0.15)  ***Favours intervention*** | OR 1.67  ***Favours intervention*** | Range: 1-4 Total:53.57  Rank:14 | Range: 1-4 Total: 64.29  Rank:8 | Range: 2-3 Total: 57.14  Rank:16 | Range: 1-3 Total: 60.71  Rank:13 | 50  Rank: 22 |
| 29 | Elective delivery in diabetic pregnant women | Induction of labour versus expectant management | Birthweight above or at the 90th percentile (not prespecified) | RR 0.43 [0.22, 0.87]  ***Favours induction*** | OR 2.69 (1.21-6.0)  ***Favours induction*** | Range: 0-4 Total: 53.57  Rank:14 | Range: 0-3 Total: 46.43  Rank:21 | Range: 0-3 Total: 46.43  Rank:21 | Range: 0-3 Total: 53.57  Rank:18 | 50  Rank: 22 |
| 30 | Psychological and pharmacological interventions for depression in patients with diabetes mellitus and depression | Pharmacological intervention vs. placebo | Depression remission: short-term | OR 2.50 (1.21, 5.15)  ***Favours intervention*** | OR 2.50 (1.21, 5.15)  ***Favours intervention*** | Range: 1-4 Total: 56.25  Rank:12 | Range: 1-3 Total: 50  Rank:19 | Range: 1-4 Total: 56.25  Rank:17 | Range: 1-3 Total: 59.38  Rank:15 | 49  Rank: 23 |
|  |  | Psychological intervention vs. usual care/waiting list control | Depression remission: medium-term | OR 2.49 (1.44 -4.32)  ***Favours intervention*** | OR 2.49 (1.44 -4.32)  ***Favours intervention*** |  |  |  |  |  |
| 31 | Targeting intensive glycaemic control versus targeting conventional glycaemic control for type 2 diabetes mellitus | Intensive glycaemic control versus conventional glycaemic control | Non-fatal myocardial infarction; best-case scenario | RR 0.38 (0.34, 0.41)  ***Favours intensive*** | OR 2.65 (2.42-2.90)  ***Favours intensive*** | Range: 1-2 Total: 43.75 Rank:19 | Range: 2-3 Total: 53.13  Rank:18 | Range: 1-3 Total: 56.25  Rank:17 | Range: 2-3 Total: 59.38  Rank:15 | 47  Rank: 24 |
| 32 | Self-monitoring of blood glucose in patients with type 2 diabetes mellitus who are not using insulin | Self-monitoring of blood glucose (SMBG) vs control (newly diagnosed patients, 12 months follow-up) | HbA1c | MD -0.52 (-0.89, -0.14)  ***Favours SMBG*** | OR 1.71  ***Favours SMBG*** | Range: 1-4 Total: 50  Rank:16 | Range: 2-4 Total: 53.13  Rank:18 | Range: 2-4 Total: 65.63  Rank:9 | Range: 2-4 Total:68.75  Rank:9 | 41  Rank: 25 |
| 33 | Complex interventions for preventing diabetic foot ulceration | Effects of more comprehensive complex interventions versus care as usual | Amputation incidence (2 years follow-up) | RR 0.30 [0.13, 0.71]  ***Favours usual care*** | OR 3.34 (1.43-7.83)  ***Favours usual care*** | Range: 1-2 Total:37.5  Rank:22 | Range: 1-2 Total:37.5  Rank:23 | Range: 2-3 Total:62.5  Rank:11 | Range: 2-4 Total: 71.88  Rank:5 | 40  Rank: 26 |
| 34 | Long-term non-pharmacological weight loss interventions for adults with type 2 diabetes mellitus | Any intervention vs usual care (F/U</=2y) | weight loss (KG) | MD -1.91 (-3.00, -0.82)  ***Favours intervention*** | OR 1.67  ***Favours intervention*** | Range: 1-2 Total: 46.88  Rank:17 | Range: 1-3 Total: 46.88  Rank:20 | Range: 1-3 Total: 50  Rank:20 | Range: 1-4 Total: 68.75  Rank:9 | 40  Rank: 27 |
| 35 | Elective delivery in diabetic pregnant women | Induction of labour versus expectant management | Caesarian section | RR 0.81 [0.52, 1.26] | OR 1.35 (0.73-2.5) | Range: 0-3 Total: 64.23  Rank:8 | Range: 0-3 Total: 53.57  Rank:17 | Range: 0-3 Total: 57.14  Rank:16 | Range: 0-3 Total: 60.71  Rank:13 | 38.75  Rank: 28 |
| 36 | Angiotensin converting enzyme inhibitors and angiotensin II receptor antagonists for preventing the progression of diabetic kidney disease | ACEi versus placebo/no treatment | All cause mortality | RR 0.91 [0.71, 1.17]  ***Favours ACEi*** | OR 1.08 (0.94-1.25)  ***Favours ACEi*** | Range: 2-4 Total: 75  Rank:3 | Range: 2-4 Total: 75  Rank:1 | Range: 3-4 Total: 83.33  Rank:2 | Range: 2-3 Total: 70.83  Rank:7 | 38.46  Rank: 29 |
| 37 | Insulin monotherapy versus combinations of insulin with oral hypoglycaemic agents in patients with type 2 diabetes mellitus | Insulin once daily versus insulin once daily plus oral antihyperglycaemic agents (OHAs) | HbA1c (change from baseline %) | MD 0.33 (0.03-0.62)  ***Favours insulin-OHA*** | OR 1.58  ***Favours insulin-OHA*** | Range: 1-2 Total: 40  Rank:21 | Range: 1-2 Total: 45  Rank:22 | Range: 0-4 Total: 60  Rank:13 | Range: 0-3 Total: 50  Rank:19 | 38.46  Rank: 29 |
| 38 | Treatment of periodontal disease for glycaemic control in people with diabetes | Scaling, root planing and oral hygiene (+/- adjunctive antibiotics) versus no/usual treatment | HbA1c after 3/4 months | MD -0.40 (-0.78, -0.01)  ***Favours intervention*** | OR 1.60  ***Favours intervention*** | Range: 1-3 Total: 57.14  Rank:11 | Range: 1-3 Total: 50  Rank:19 | Range: 1-4 Total: 64.29  Rank:10 | Range: 1-4 Total: 71.43  Rank:6 | 23.81  Rank: 30 |

**HIV/AIDS**

|  | Review Title | Intervention | Outcome | Original effect size (from Cochrane review) | Effect Size (converted to OR for comparison) | Feasibility^[[9]](#footnote-9)^ | Deliverability ^[[10]](#footnote-10)^ | Universality ^[[11]](#footnote-11)^ | Effect on Equity^[[12]](#footnote-12)^ | Overall Rating (%) |
| --- | --- | --- | --- | --- | --- | --- | --- | --- | --- | --- |
| 1 | Prevention of diarrhoea in children with HIV infection or exposure to maternal HIV infection | vitamin A vs placebo | mortality - all cause | Original RR 0.5 (0.31-0.79) ***Favours vit A*** | OR 1.89 (0.96-3.70) ***Favours vit A*** | Range: 2-4 Total: 87.5  Rank: 1 | Range: 2-4 Total: 87.5  Rank: 1 | Range: 3-4 Total: 91.67  Rank: 2 | Range: 2-4 Total: 87.5  Rank: 1 | 96.25  Rank: 1 |
| 2 | Behavioral interventions for improving contraceptive use among women living with HIV | family planning intervention vs usual care | non-condom contraceptive use | OR 6.40 (5.37-7.62) ***Favours intervention*** | OR 6.40 (5.37-7.62) ***Favours intervention*** | Range: 2-4 Total: 75  Rank: 4 | Range: 2-4 Total: 75  Rank: 4 | Range: 2-4 Total: 79.17  Rank: 6 | Range: 2-4 Total: 83.33  Rank: 2 | 96.25  Rank: 1 |
| 3 | Behavioral interventions to reduce the transmission of HIV infection among sex workers and their clients in low‐ and middle‐income countries | promotion of female and male condom vs promotion of male condom | consistent female condom use by FSW at 3 month | Original RR 0.12(0.09-0.17) ***favours promotion of both*** | OR >10 ***Favours promotion of both*** | Range: 2-4 Total: 75  Rank: 4 | Range: 2-4 Total: 83.33  Rank: 2 | Range: 2-4 Total: 83.33  Rank: 4 | Range: 2-4 Total: 87.5  Rank: 1 | 91.25  Rank: 2 |
| 4 | Behavioral interventions for improving contraceptive use among women living with HIV | Family planning counseling (HIV+ women) versus comparison (HIV- women) | discontinued use of hormonal contraceptive | OR 2.52 (1.53-4.14) ***Favours HIV-*** | OR 2.52 (1.53-4.14) ***Favours HIV-*** | Range: 2-4 Total: 70.83  Rank: 5 | Range: 2-4 Total: 70.83  Rank: 5 | Range: 2-4 Total: 70.83  Rank: 8 | Range: 0-4 Total: 58.33  Rank: 10 | 86.25  Rank: 3 |
| 5 | Cotrimoxazole prophylaxis for opportunistic infections in adults with HIV | Cotrimoxazole vs control | death | Original RR 0.69 (0.55-0.87 ***Favours Cotrimoxazole*** | OR 1.57 (1.18-2.08) ***Favours Cotrimoxazole*** | Range: 2-4 Total: 87.5  Rank: 1 | Range: 3-4 Total: 75  Rank: 4 | Range: 3-4 Total: 95.83  Rank: 1 | Range: 0-4 Total: 79.17  Rank: 3 | 86.25  Rank: 3 |
| 6 | Micronutrient supplementation in pregnant women with HIV infection | Multivitamins vs no multivitamins | fetal death | Original RR 0.61 (0.39-0.94) ***Favours multivitamin*** | OR 1.71 (1.07-2.74) ***Favours multivitamin*** | Range: 2-4 Total: 79.17  Rank: 3 | Range: 1-4 Total: 75  Rank: 4 | Range: 2-4 Total: 87.5  Rank: 3 | Range: 2-4 Total: 79.17  Rank: 3 | 84.44  Rank: 4 |
| 7 | Antiretroviral therapy (ART) for treating HIV infection in ART‐eligible pregnant women | zidovudine (AZT), lamivudine (3TC) and lopinavir/ritonavir (LPV-r) starting at 28-36 weeks gestation vs short course regimen | infant HIV transmission or death at 12 months | Original RR 0.64 (0.44-0.92) ***Favours AZT/3TC/LPV-r*** | OR 1.68 (1.10-2.57) ***Favours AZT/3TC/LPV-r*** | Range: 2-4 Total: 75  Rank: 4 | Range: 2-4 Total: 70.83  Rank: 5 | Range: 2-4 Total: 83.33  Rank: 4 | Range: 3-4 Total: 87.5  Rank: 1 | 82.5  Rank: 5 |
| 8 | Deworming helminth co‐infected individuals for delaying HIV disease progression | Antihelminthic treatment | change in log10 HIV-1 RNA | Original MD -0.18 (-0.33, -0.03) ***Favours treatment*** | OR 1.58 ***Favours treatment*** | Range: 2-4 Total: 87.5  Rank: 1 | Range: 2-4 Total: 87.5  Rank: 1 | Range: 2-4 Total: 87.5  Rank: 3 | Range: 0-4 Total: 66.67  Rank: 7 | 81.25  Rank: 6 |
| 9 | Interventions for the prevention and management of oropharyngeal candidiasis associated with HIV infection in adults and children | Fluconazole  Vs no treatment | clinical episode of oropharyngeal candidiasis (adults) | RR 0.16 (0.08-0.34) ***Favours fluconazole*** | OR >10 ***Favours fluconazole*** | Range: 2-4 Total: 70.83  Rank: 5 | Range: 1-3 Total: 62.5  Rank: 8 | Range: 1-4 Total: 70.83  Rank: 8 | Range: 1-4 Total: 58.33  Rank: 10 | 81.25  Rank: 6 |
| 10 | Behavioral interventions for improving contraceptive use among women living with HIV | Integrated family planning services: enhanced versus basic | modern contraceptive method use | OR 2.48 (1.31-4.72) ***Favours enhanced*** | OR 2.48 (1.31-4.72) ***Favours enhanced*** | Range: 2-3 Total: 62.5  Rank: 7 | Range: 2-3 Total: 58.33  Rank: 10 | Range: 2-4 Total: 79.17  Rank: 6 | Range: 2-4 Total: 75  Rank: 4 | 80  Rank: 7 |
| 11 | Interventions to reduce risky sexual behaviour for preventing HIV infection in workers in occupational settings | on site testing and counseling vs. voucher for off-site VCTS | uptake of testing | RR 14.00 (11.75-16.68) ***Favours on-site*** | OR >10 ***Favours on-site*** | Range: 2-4 Total: 70.83  Rank: 5 | Range: 3-4 Total: 83.33  Rank: 2 | Range: 2-4 Total: 70.83  Rank: 8 | Range: 2-3 Total: 62.5  Rank: 9 | 80  Rank: 7 |
| 12 | Micronutrient supplementation in children and adults with HIV infection | vitamin A – children vs. placebo | all cause mortality | Original RR 0.55(0.37-0.82) ***Favours experimental*** | OR 2.30 (1.33-3.98) ***Favours experimental*** | Range: 1-4 Total: 79.17  Rank: 3 | Range: 2-4 Total: 83.33  Rank: 2 | Range: 2-4 Total: 87.5  Rank: 3 | Range: 0-4 Total: 75  Rank: 4 | 77.78  Rank: 8 |
| 13 | Antiretroviral therapy for prevention of HIV transmission in HIV-discordant couples | Treated with ART vs. not treated with ART | Incident HIV infection (observational studies) | RR 0.58 (0.35-0.96) ***Favours treatment*** | OR 1.34 (0.51-3.55) ***Favours treatment*** | Range: 2-4 Total: 75  Rank: 4 | Range: 2-4 Total: 70.83  Rank: 5 | Range: 2-4 Total: 87.5  Rank: 3 | Range: 2-4 Total: 79.17  Rank: 3 | 76.25  Rank: 9 |
|  |  |  | Incident HIV infection (observational studies - (sensitivity analysis – 2 studies removed) | RR 0.36 (0.17-0.75) ***Favours treatment*** | OR 1.34 (0.51-3.55) ***Favours treatment*** |  |  |  |  |  |
| 14 | Optimisation of antiretroviral therapy in HIV-infected children under 3 years of age | early vs deferred ART | Mortality | Original HR 0.36 (0.18-0.74) ***Favours deferred*** | OR 4.16(1.88-9.19) ***Favours deferred*** | Range: 0-4 Total: 62.5  Rank: 8 | Range: 0-4 Total: 62.5  Rank: 8 | Range: 3-4 Total: 87.5  Rank: 3 | Range: 0-4 Total: 56.25  Rank: 11 | 71.05  Rank: 10 |
| 15 | Behavioral interventions to reduce the transmission of HIV infection among sex workers and their clients in low‐ and middle‐income countries | Social and cognitive behavioural intervention | HIV and STI incidence at 6 mos. | RR 0.12 (0.01-2.22) ***Favours social cognitive theory intervention*** | OR 7.48 (0.39–142) ***Favours social cognitive theory intervention*** | Range: 2-3 Total: 65  Rank: 7 | Range: 2-4 Total: 70  Rank: 6 | Range: 1-4 Total: 65  Rank: 10 | Range: 1-4 Total: 65 Rank: 8 | 70  Rank: 11 |
| 16 | Treatment of severe or progressive Kaposi's sarcoma in HIV-infected adults | HAART + ABV versus HAART alone (RCT) | Overall response (complete and partial) including number of lesions, size, oedema | RR 1.78 (1.16-2.72) ***Favours HAART alone*** | OR 3.16 (1.4, 7.15) ***Favours HAART alone*** | Range: 1-4 Total: 75  Rank: 4 | Range: 1-4 Total: 70.83  Rank: 5 | Range: 0-4 Total: 50  Rank: 15 | Range: 2-4 Total: 54.17  Rank: 12 | 70  Rank: 11 |
| 17 | Antiretroviral pre‐exposure prophylaxis (PrEP) for preventing HIV in high‐risk individuals | tenofovir disoproxil fumarate (TDF) plus emtricitabine (FTC) versus placebo | HIV infection | Original RR 0.49 (0.28-0.85) ***Favours TDF+FTC*** | OR 2.0 (1.66-2.40) ***Favours TDF+FTC*** | Range: 1-3 Total: 54.17  Rank: 11 | Range: 2-3 Total: 62.5  Rank: 8 | Range: 2-4 Total: 75  Rank: 7 | Range: 2-4 Total: 75  Rank: 4 | 68.75  Rank: 12 |
| 18 | Optimisation of antiretroviral therapy in HIV-infected children under 3 years of age | NVP based vs LPV/r based ART | Treatment failure | Original HR 2.01 (1.47-2.77) ***Favours NVP*** | OR 2.88 (1.69-4.91) ***Favours NVP*** | Range: 2-4 Total: 75  Rank: 4 | Range: 1-4 Total: 70  Rank: 6 | Range: 1-4 Total: 75  Rank: 7 | Range: 2-4 Total: 70  Rank: 6 | 68.42  Rank: 13 |
| 19 | Optimal time for initiation of antiretroviral therapy in asymptomatic, HIV ‐infected, treatment‐naive adults | early vs. standard or deferred ART | death | RR 0.26 (0.11-0.62) ***Favours early treatment*** | OR 4.25 (1.72-10.48) ***Favours early treatment*** | Range: 2-4 Total: 66.67  Rank: 6 | Range: 1-4 Total: 75  Rank: 4 | Range: 2-4 Total: 83.33  Rank: 4 | Range: 0-4 Total: 66.67  Rank: 7 | 67.78  Rank: 14 |
| 20 | Male circumcision for prevention of homosexual acquisition of HIV in men | male circumcision | HIV infection | Original OR 0.86 (0.70-1.06) ***Favours circumcision*** | OR 1.16 (0.94-1.43) ***Favours circumcision*** | Range: 1-2 Total: 37.5  Rank: 14 | Range: 1-3 Total: 45.83  Rank: 13 | Range: 1-4 Total: 62.5  Rank: 11 | Range: 1-4 Total: 66.67  Rank: 7 | 67.5  Rank: 15 |
|  |  |  | HIV infection (subgroup: mainly insertive anal sex) | Original OR0.27 (0.17-0.44) ***Favours circumcision*** | OR 3.70 (2.72-5.88) ***Favours circumcision*** |  |  |  |  |  |
| 21 | Optimal monitoring strategies for guiding when to switch first‐line antiretroviral therapy regimens for treatment failure in adults and adolescents living with HIV in low‐resource settings | Clinical Monitoring vs Immunologic Monitoring and Clinical Monitoring | mortality | Original HR 1.35 (1.12-1.63) ***Favours combination*** | OR 1.65 (1.12-2.46) ***Favours combination*** | Range: 2-4 Total: 75  Rank: 4 | Range: 2-4 Total: 79.17  Rank: 3 | Range: 2-4 Total: 91.67  Rank: 2 | Range: 1-4 Total: 83.33  Rank: 2 | 65.56  Rank: 16 |
| 22 | Tenofovir or zidovudine in three‐drug combination therapy with one nucleoside reverse transcriptase inhibitor and one non‐nucleoside reverse transcriptase inhibitor for initial treatment of HIV infection in antiretroviral‐naïve individuals | Tenofovir (TDF) vs zidovudine (AZT) | Virologic response | RR 2.04 (0.17-24.84) ***Favours TDF*** | OR 1.35 (0.94-1.96) ***Favours TDF*** | Range: 3-4 Total: 83.33  Rank: 2 | Range: 2-4 Total: 87.5  Rank: 1 | Range: 3-4 Total: 87.5  Rank: 3 | Range: 0-4 Total: 75  Rank: 4 | 65  Rank: 17 |
|  |  |  | immunological response | MD 32.0 (13.86-50.14) ***Favours TDF*** | OR 1.74 ***Favours TDF*** |  |  |  |  |  |
| 23 | Decentralising HIV treatment in lower‐ and middle‐income countries | Partial decentralization - ART in hospital maintained in health centre (vs. hospital care) | death at 12 months | Original RR 0.34 (0.13-0.87) ***Favours decentral.*** | OR 1.26 (1.17-1.37)  ***Favours decentral.*** | Range: 1-4 Total: 66.67  Rank: 6 | Range: 2-4 Total: 75  Rank: 4 | Range: 3-4 Total: 83.33  Rank: 4 | Range: 0-4 Total: 70.83  Rank: 5 | 63.75  Rank: 18 |
| 24 | Mass media interventions for promoting HIV testing | leaflets  vs control | uptake of testing (RCT) | OR 9.12 (6.85-12.15) ***Favours leaflet*** | OR 9.12 (6.85-12.15) ***Favours leaflet*** | Range: 2-4 Total: 83.33  Rank: 2 | Range: 3-4 Total: 87.5  Rank: 1 | Range: 3-4 Total: 91.67  Rank: 2 | Range: 0-4 Total: 58.33  Rank: 10 | 61.11  Rank: 19 |
| 25 | Cotrimoxazole prophylaxis for opportunistic infections in adults with HIV | cotrimoxazole and leucovorin vs control | PCP infection | Original RR 0.31 (0.13-0.74) ***Favours cotrimoxazole and leucovorin*** | OR 5.71 (1.72-18.94) ***Favours cotrimoxazole and leucovorin*** | Range: 0-4 Total: 54.17  Rank: 11 | Range: 2-4 Total: 70.83  Rank: 5 | Range: 1-4 Total: 70.83  Rank: 8 | Range: 0-4 Total: 54.17  Rank: 12 | 58.89  Rank: 20 |
| 26 | Three‐ or four‐ versus two‐drug antiretroviral maintenance regimens for HIV infection | protease inhibitor-excluding maintenance regimens vs continued induction | loss of viral suppression | OR 5.99 (3.83-9.38) ***Favours continued induction*** | OR 5.99 (3.83-9.38) ***Favours continued induction*** | Range: 0-4 Total: 58.33  Rank: 10 | Range: 2-3 Total: 62.5  Rank: 8 | Range: 0-4 Total: 66.67  Rank: 9 | Range: 0-4 Total: 54.17  Rank: 12 | 58.14  Rank: 21 |
| 27 | Three‐ or four‐ versus two‐drug antiretroviral maintenance regimens for HIV infection | antiretroviral-sparing maintenance therapy vs. continued induction therapy | loss of viral suppression | OR 4.63 (3.08-6.96) ***Favours continued induction*** | OR 4.63 (3.08-6.96) ***Favours continued induction*** | Range: 0-4 Total: 58.33  Rank: 10 | Range: 0-4 Total: 62.5  Rank: 8 | Range: 0-4 Total: 66.67  Rank: 9 | Range: 0-4 Total: 54.17  Rank: 12 | 58.14  Rank: 21 |
| 28 | Male circumcision for prevention of heterosexual acquisition of HIV in men | Circumcision | HIV incidence 12-24 | RR 0.46 (0.34-0.62) ***Favours circumcision*** | OR 2.02 (1.21-3.38) ***Favours circumcision*** | Range: 1-3 Total: 45.83  Rank: 13 | Range: 1-3 Total: 54.17  Rank: 11 | Range: 1-4 Total: 79.17  Rank: 6 | Range: 0-4 Total: 54.17  Rank: 12 | 56.75  Rank: 22 |
| 29 | Behavioral interventions for improving contraceptive use among women living with HIV | Informational video +discussion (HIV+ vs HIV- women) | incident pregnancy after intervention | Original OR 0.39 (0.23-0.68) ***Favours HIV+*** | OR 2.56 (1.47-4.35) ***Favours HIV+*** | Range: 1-3 Total: 54.17  Rank: 11 | Range: 2-4 Total: 58.33  Rank: 10 | Range: 1-4 Total: 58.33  Rank: 12 | Range: 1-4 Total: 58.33  Rank: 10 | 56.58  Rank: 23 |
| 30 | Progressive resistive exercise interventions for adults living with HIV/AIDS | progressive resistance exercise or combined progressive resistance and aerobic vs non-exercising control | weight measures (kg) | Original MD 3.54 (2.21-4.87) ***Favours exercise*** | OR 9.68 ***Favours exercise*** | Range: 1-4 Total: 54.17  Rank: 11 | Range: 1-4 Total: 54.17  Rank: 11 | Range: 1-4 Total: 66.67  Rank: 9 | Range: 1-4 Total: 62.5  Rank: 9 | 55  Rank: 24 |
| 31 | Three‐ or four‐ versus two‐drug antiretroviral maintenance regimens for HIV infection | indinavir-including maintenance regimens vs. vs. zidovudine-lamivudine-indinavir continued induction therapy | loss of viral suppression | OR 3.88 (2.28-6.61) ***Favours continued induction*** | OR 3.88 (2.28-6.61) ***Favours continued induction*** | Range: 0-4 Total: 58.33  Rank: 10 | Range: 0-4 Total: 62.5  Rank: 8 | Range: 0-4 Total: 66.67  Rank: 9 | Range: 0-4 Total: 54.17  Rank: 12 | 52.63  Rank: 25 |
| 32 | Optimisation of antiretroviral therapy in HIV-infected children under 3 years of age | switch to NVP vs continue on LPV/r | virological failure | Original HR 10.19 (2.36-43.94) ***Favours switch (NVP)*** | OR 5.16 (1.16-22.92) ***Favours switch(NVP)*** | Range: 2-4 Total: 75  Rank: 4 | Range: 2-4 Total: 75  Rank: 4 | Range: 2-4 Total: 80  Rank: 5 | Range: 2-4 Total: 70  Rank: 6 | 50  Rank: 26 |
| 33 | Treatment of latent tuberculosis infection in HIV infected persons | isoniazid plus rifampicin vs. placebo | death (all cause) | Original RR 0.69 (0.50-0.95) ***Favours isoniazid + rifampicin*** | OR 1.52 (1.06-2.18) ***Favours isoniazid + rifampicin*** | Range: 2-3 Total: 58.33  Rank: 10 | Range: 2-3 Total: 66.67  Rank: 7 | Range: 2-4 Total: 83.33  Rank: 4 | Range: 0-4 Total: 66.67  Rank: 7 | 47.5  Rank: 27 |
| 34 | Home‐based HIV voluntary counselling and testing (VCT) for improving uptake of HIV testing | clinic versus optional location | post-test counseled and received test result | RR 4.74 (3.62-6.21 ***Favours optional location*** | OR 9.46 (6.67-13.41) ***Favours optional location*** | Range: 1-4 Total: 58.33  Rank: 10 | Range: 2-4 Total: 75  Rank: 4 | Range: 2-3 Total: 83.33  Rank: 4 | Range: 1-5 Total: 79.17  Rank: 3 | 46.67  Rank: 28 |
| 35 | Treatment of severe or progressive Kaposi's sarcoma in HIV-infected adults <http://onlinelibrary.wiley.com/doi/10.1002/14651858.CD003256/abstract> | HAART + ABV versus HAART alone (RCT) | Progressive disease | RR 0.10 (0.01-0.75)***f Favours HAART+chemo*** | OR >10  ***Favours HAART+chemo*** | Range: 1-3 Total: 50  Rank: 12 | Range: 1-3 Total: 50  Rank: 12 | Range:0-2 Total:29.17  Rank: 16 | Range: 0-3 Total: 37.5  Rank: 17 | 46.25  Rank: 29 |
| 36 | Decentralising HIV treatment in lower‐ and middle‐income countries | Full decentralization - ART in started and maintained in health centre (vs hospital care) | Lost to care at 12 months | Original RR 0.30 (0.17-0.54) ***Favours decentral.*** | OR 2.74 (2.60-2.88) ***Favours decentral*** | Range: 1-3 Total: 50  Rank: 12 | Range: 0-4 Total: 54.17  Rank: 11 | Range: 1-4 Total: 75  Rank: 7 | Range: 0-4 Total: 62.5  Rank: 9 | 45.56  Rank: 30 |
| 37 | Adjunctive corticosteroids for Pneumocystis jiroveci pneumonia in patients with HIV ‐infection | adjunctive corticosteroids  vs no such treatment | death at 3-4 months | Original RR 0.68 (0.5-0.94) ***Favours adjunctive corticosteroids*** | OR 2.15 (1.34-3.45) ***Favours adjunctive corticosteroids*** | Range: 1-4 Total: 62.5  Rank: 8 | Range: 1-3 Total: 58.33  Rank: 10 | Range: 1-4 Total: 58.33  Rank: 12 | Range: 0-4 Total: 45.83  Rank: 14 | 43.75  Rank: 31 |
| 38 | Interventions for the prevention of mycobacterium avium complex in adults and children with HIV | Clarithromycin vs placebo | development of MAC disease | Original RR 0.35 (0.21-0.58) ***Favours Clarithromycin*** | OR 3.19 (1.84-5.51) ***Favours Clarithromycin*** | Range: 2-3 Total: 75  Rank: 4 | Range: 2-3 Total: 75  Rank: 4 | Range: 2-4 Total: 83.33  Rank: 4 | Range: 1-4 Total: 66.67  Rank: 7 | 41.25  Rank: 32 |
| 39 | Adjunctive corticosteroids for Pneumocystis jiroveci pneumonia in patients with HIV ‐infection | Corticosteroids vs no such treatment | death at 3-4 months | Original RR 0.68 (0.5-0.94) ***Favours corticosteroids*** | OR 1.68 (1.09-2.58) ***Favours corticosteroids*** | Range: 1-3 Total: 54.17  Rank: 11 | Range: 1-3 Total: 54.17  Rank: 11 | Range: 1-4 Total: 58.33  Rank: 12 | Range: 0-4 Total: 45.83  Rank: 14 | 38.75  Rank: 33 |
| 40 | Interventions for the prevention of mycobacterium avium complex in adults and children with HIV | Azithromycin vs placebo | development of MAC disease | Original RR 0.37 (0.19-0.74) ***Favours Azithromycin*** | OR 2.93 (1.36-6.31) ***Favours Azithromycin*** | Range: 2-4 Total: 70.83  Rank: 5 | Range: 2-4 Total: 70.83  Rank: 5 | Range: 2-4 Total: 75  Rank: 7 | Range: 2-4 Total: 62.5  Rank: 9 | 38.75  Rank: 33 |
| 41 | Interventions for the prevention of mycobacterium avium complex in adults and children with HIV | Rifabutin plus azithromycin vs. rifabutin | development of mycobacterium avium complex (MAC) disease | Original RR 0.35 (0.35-0.59) ***Favours Rifabutin plus azithromycin*** | OR 3.38 (1.90-6.00) ***Favours Rifabutin plus azithromycin*** | Range: 1-3 Total: 54.17  Rank: 11 | Range: 1-3 Total: 54.17  Rank: 11 | Range: 2-4 Total: 54.17  Rank: 14 | Range: 0-4 Total: 45.83  Rank: 14 |  |
| 42 | Interventions for the prevention of mycobacterium avium complex in adults and children with HIV | Rifabutin vs placebo | development of MAC disease | Original RR 0.48 (0.35-0.67) ***Favours Rifabutin*** | OR 2.30 (1.60-3.32) ***Favours Rifabutin*** | Range: 1-4 Total: 58.33  Rank: 10 | Range: 1-4 Total: 58.33  Rank: 10 | Range: 1-4 Total: 62.5  Rank: 11 | Range: 0-4 Total: 50 Rank: 13 | 32.5  Rank: 34 |
| 43 | Zidovudine (AZT) versus AZT plus didanosine (ddI) versus AZT plus zalcitabine (ddC) in HIV infected adults | Zidovudine (AZT) plus didanosine (ddI) vs AZT alone | Death | Original RR 0.72 (0.64-0.82) ***Favours AZT+ddl*** | OR 1.41 (1.22-1.63) ***Favours AZT+ddl*** | Range: 0-3 Total: 50  Rank: 12 | Range: 0-3 Total: 50  Rank: 12 | Range: 0-4 Total: 58.33  Rank: 12 | Range: 0-4 Total: 50  Rank: 13 | 30.26  Rank: 35 |
| 44 | Interventions for the prevention of mycobacterium avium complex in adults and children with HIV | Rifabutin plus Clarithromycin vs. rifabutin | development of MAC disease | Original RR 0.44 (0.29-0.69) ***Favours Rifabutin plus Clarithromycin*** | OR2.48 (1.53-4.03) ***Favours Rifabutin plus Clarithromycin*** | Range: 1-3 Total: 45.83  Rank: 13 | Range: 1-3 Total: 50  Rank: 12 | Range: 0-4 Total: 50  Rank: 15 | Range: 0-4 Total: 45.83  Rank: 14 | 30  Rank: 36 |
| 45 | Anabolic steroids for the treatment of weight loss in HIV ‐infected individuals | anabolic steroid  vs placebo | change in body weight | MD 1.13 (0.29-1.96)  ***Favours steroid*** | OR 1.76 ***Favours steroid*** | Range: 1-3 Total: 45.83  Rank: 13 | Range: 1-4 Total: 50  Rank: 12 | Range: 1-4 Total: 54.17  Rank: 14 | Range: 0-4 Total: 41.67  Rank: 15 | 28.75  Rank: 37 |
| 46 | Zidovudine (AZT) versus AZT plus didanosine (ddI) versus AZT plus zalcitabine (ddC) in HIV infected adults | Zidovudine (AZT) plus didanosine (ddI) vs AZT plus zalcitabine (ddC) | Death | Original RR 0.86 (0.77-0.96) ***Favours AZT+ddl*** | OR 1.18 (1.03-1.34) ***Favours AZT+ddl*** | Range: 2-3 Total: 60  Rank: 9 | Range: 0-3 Total: 60  Rank: 9 | Range: 0-4 Total: 55  Rank: 13 | Range: 0-4 Total: 40  Rank: 16 | 25  Rank: 38 |
| 47 | Zidovudine (AZT) versus AZT plus didanosine (ddI) versus AZT plus zalcitabine (ddC) in HIV infected adults | Zidovudine (AZT) plus zalcitabine (ddC) vs AZT alone | Death | Original RR 0.87 (0.77-0.97) ***Favours AZT+ddC*** | OR 1.17 (1.03-1.34) ***Favours AZT+ddC*** | Range: 2-3 Total: 60  Rank: 9 | Range: 0-3 Total: 40  Rank: 14 | Range: 0-4 Total: 50  Rank: 15 | Range: 0-4 Total: 35  Rank: 18 | 22.22  Rank: 39 |

Malaria

|  | Review Title | Intervention | Trial Endpoint | Original Effect size (from Cochrane review) | Effect Size | Feasibility^[[13]](#footnote-13)^ | Deliverability ^[[14]](#footnote-14)^ | Universality ^[[15]](#footnote-15)^ | Effect on Equity^[[16]](#footnote-16)^ | Overall Rating (%) |
| --- | --- | --- | --- | --- | --- | --- | --- | --- | --- | --- |
| 1 | Unit-dose packaged drugs for treating malaria | Tablets in sectioned polythene bags versus bottled syrup  (control: bottled syrup – same drug as intervention) | Treatment adherence (measured by interview) | RR 2.15 [ 1.76, 2.61  ***Favours bags*** | OR 7.00 (3.39-14.48)  ***Favours bags*** | Range: 3-4  Total: 87.5  Rank:1 | Range: 2-4  Total: 79.17  Rank:1 | Range: 0-4  Total: 58.33  Rank:8 | Range: 3-4  Total: 91.67  Rank:2 | 84.06  Rank: 1 |
| 2 | Artemisinin-based combination therapy for treating uncomplicated malaria | Artemether-lumefantrine (AL) vs amodiaquine plus sulfadoxine-pyrimethamine  (control:amodiaquine + sulfadoxine pyrimethimine) | Total Failure (P. falciparum) Day 28 PCR adjusted (East Africa) | Original RR 0.12 (0.06-0.24)  ***Favours AL*** | OR >10  ***Favours AL*** | Range: 3-4  Total: 79.17  Rank:2 | Range:1-4  Total: 70.83  Rank:2 | Range: 3-4  Total: 83.33  Rank:1 | Range: 0-4  Total: 70.83  Rank:4 | 79.38  Rank: 2 |
| 3 | Artemisinin-based combination therapy for treating uncomplicated malaria | Dihydroartemisinin-piperaquine vs Artesunate plus mefloquine  (control : artesunate plus mefloequine (AS+MQ)) | Total Failure (P. falciparum) Day 63 PCR unadjusted: South America | RR 6.19 (1.40-27.35)  ***Favours AS+MQ*** | OR 6.49 (1.44-29.36)  ***Favours AS+MQ*** | Range: 1-4  Total: 70.83  Rank:4 | Range: 1-4  Total: 62.5  Rank:4 | Range: 2-4  Total: 75  Rank:3 | Range: 1-4  Total: 62.5  Rank:6 | 79.38  Rank:2 |
| 4 | Home- or community-based programmes for treating malaria | Home- or community-based programmes vs standard care | All-Cause Mortality (at follow up) | RR 0.58[0.44,0.77]  ***Favours Home- or community-based care*** | OR 1.76 (1.11-2.81)  ***Favours Home- or community-based care*** | Range: 1-4  Total: 50  Rank:10 | Range: 1-4  Total: 54.17  Rank:7 | Range: 2-4  Total: 75  Rank:3 | Range: 3-4  Total: 95.83  Rank:1 | 77.81  Rank:3 |
| 5 | Intermittent preventive treatment regimens for malaria in HIV-positive pregnant women | Monthly sulfadoxine-pyrimethamine (SP) compared to standard 2-dose SP  (control: standard 2-dose SP) | Maternal peripheral parasitaemia (at delivery) | RR 0.25 (0.14 to 0.43)  ***Favours 2 dose SP*** | OR 5.36 (1.94-14.80)  ***Favours 2 dose SP*** | Range: 1-4  Total: 65  Rank:6 | Range: 1-4  Total: 60  Rank:3 | Range: 1-3  Total: 60  Rank:7 | Range: 3  Total: 75  Rank:3 | 76.88  Rank:4 |
| 6 | Intermittent preventive treatment for malaria in children living in areas with seasonal transmission | Intermittent Preventive Treatment of malaria (IPTc) versus placebo  (control: placebo) | Severe malaria – during intervention | Original RR 0.27 (0.1 to 0.76)  ***Favours IPTc*** | OR 1.01 (0.33-2.0)  ***Favours IPTc*** | Range: 2-3  Total: 54.17  Rank:9 | Range: 1-3  Total: 50  Rank:8 | Range: 2-4  Total: 66.67  Rank:5 | Range: 2-4  Total: 75  Rank:3 | 74.69  Rank:5 |
| 7 | Artemisinin-based combination therapy for treating uncomplicated malaria | DHA-P vs Amodiaquine plus sulfadoxine-pyrimethamine  (control: amodiaquine + sulfadoxine-pyrimethamine) | Effectiveness: Total Failure (P. falciparum) PCR adjusted: Day 28: | Original RR 0.32 (0.16-0.64)  ***Favours DHA-P*** | OR 3.63 (1.96-6.72)  ***Favours DHA-P*** | Range: 2-4  Total: 75  Rank:3 | Range: 1-4  Total: 66.67  Rank:3 | Range: 2-4  Total: 75  Rank:3 | Range: 0-4  Total: 66.67  Rank:5 | 73.44  Rank:6 |
| 8 | Artemisinin-based combination therapy for treating uncomplicated malaria | Dihydroartemisinin-piperaquine vs Artesunate plus amodiaquine  (control : Artesunate plus amodiaquine) | Total Failure (P. falciparum) Day 42 PCR adjusted (Asia) | RR  0.10 [ 0.01, 0.81 ]  ***Favours DHA-P*** | OR >10  ***Favours DHA-P*** | Range: 2-4  Total: 70.83  Rank:4 | Range: 1-4  Total: 66.67  Rank:3 | Range: 2-4  Total: 70.83  Rank:4 | Range: 0-4  Total: 62.5  Rank:6 | 73.21  Rank:7 |
| 9 | Artemisinin-based combination therapy for treating uncomplicated malaria | Artemether-lumefantrine (AL) vs Artesunate plus sulfadoxine-pyrimethamine  (control: Artesunate plus sulfadoxine-pyrimethamine) | Total Failure (P. falciparum) Day 42 PCR adjusted (Oceania) | Original RR 0.33 (0.13-0.86)  ***Favours AL*** | OR 3.50 (1.22-10.03)  ***Favours AL*** | Range: 3-4  Total: 79.17  Rank:2 | Range: 1-4  Total: 66.67  Rank:3 | Range: 1-4  Total: 66.67  Rank:5 | Range: 1-4  Total: 62.5  Rank:6 | 72.19  Rank:8 |
| 10 | Artemisinin-based combination therapy for treating uncomplicated malaria | Artesunate plus amodiaquine vs Amodiaquine plus sulfadoxine-pyrimethamine (AQSP)  (control: amodiaquine plus sulfadoxine-pyrimethamine (AQSP)) | Total Failure (P. falciparum) Day 28 PCR unadjusted (other) | Original RR 3.12 (1.05-9.25)  ***Favours AQSP*** | OR 3.52 (1.08-11.44)  ***Favours AQSP*** | Range: 2-4  Total: 75  Rank:3 | Range: 1-4  Total: 62.5  Rank:4 | Range: 2-4  Total: 70.83  Rank:4 | Range: 1-4  Total: 62.5  Rank:6 | 70  Rank: 9 |
| 11 | Artemisinin-based combination therapy for treating uncomplicated malaria | Dihydroartemisinin-piperaquine vs Artemether-lumefantrine  (control: artemether-lumefantrine (AL)) | Total Failure (P. falciparum) Day 28 PCR unadjusted (Africa) | Original RR 0.12 (0.05-0.32)  ***Favours DHA-P*** | OR >10  ***Favours DHA-P*** | Range: 1-4  Total: 79.17  Rank:2 | Range: 1-4  Total: 62.5  Rank:4 | Range: 2-4  Total: 79.17  Rank:2 | Range: 0-4  Total: 62.5  Rank:6 | 70  Rank: 9 |
| 12 | Dihydroartemisinin-piperaquine for treating uncomplicated Plasmodium falciparum malaria | Dihydroartemisinin-piperaquine vs. Artemether-lumefantrine | treatment failure day 28 PCR adjusted (Africa) | RR 0.42 (0.29 to 0.62) ***Favours DHA-P*** | OR 2.34 (1.60-3.42) ***Favours DHA-P*** | Range: 1-4  Total: 75  Rank:3 | Range: 1-4  Total: 62.5  Rank:4 | Range: 2-4  Total: 79.17  Rank:2 | Range: 1-4  Total: 66.67  Rank:5 | 70  Rank: 9 |
| 13 | Drugs for treating uncomplicated malaria in pregnant women  <http://onlinelibrary.wiley.com/doi/10.1002/14651858.CD004912.pub3/abstract> | Artesunate plus sulfadoxine-pyrimethamine (AS+SP) vs sulfadoxine-pyrimethamine (SP)  (control SP) | Treatment failure at delivery or day 40 (excludes new infections, detected by PCR) | Original OR 0.15 (0.04-0.59)  ***Favours AS+SP*** | OR 6.67 (1.69-25.0)  ***Favours AS+SP*** | Range: 2-4  Total: 75  Rank:3 | Range: 1-3  Total: 54.17  Rank:7 | Range: 0-3  Total: 45.83  Rank:11 | Range: 1-3  Total: 54.17  Rank:8 | 59.38  Rank: 10 |
| 14 | Drugs for treating uncomplicated malaria in pregnant women | Azithromycin plus sulfadoxine-pyrimethamine (AZM+SP) vs sulfadoxine-pyrimethamine (SP)  (control: SP) | Treatment failure at delivery or day 40 (excludes new infections, detected by PCR) | Original RR 0.27 (0.10-0.76)  ***Favours AZM+SP*** | OR 5.11 (1.51-17.29)  ***Favours AZM+SP*** | Range: 1-4  Total: 75  Rank:3 | Range: 1-3  Total: 58.33  Rank:6 | Range:0-3  Total: 45.83  Rank:11 | Range: 1-3  Total: 50  Rank:9 | 53.13  Rank: 11 |
| 15 | Drugs for treating uncomplicated malaria in pregnant women | Amodiaquine plus sulfadoxine-pyrimethamine (AQ+SP) vs chloroquine (CQ)  (Control: CQ) | Treatment failure at day 28 | Original RR 0.08 (0.03-0.19)  ***Favours AQ+SP*** | OR>10  ***Favours AQ+SP*** | Range: 1-4  Total: 62.5  Rank:7 | Range: 1-3 Total: 50  Rank:8 | Range: 0-3  Total: 37.5  Rank:13 | Range: 0-3  Total: 50  Rank:9 | 49.38  Rank: 12 |
| 16 | High first dose quinine regimen for treating severe malaria | High first (loading) dose compared with no loading dose  (control: no loading dose) | Death | RR 0.62 (0.19-2.04)  ***Favours loading dose*** | OR 1.72 0.48-6.17) | Range: 2-3  Total: 58.33  Rank:8 | Range: 0-3  Total: 58.33  Rank:6 | Range: 0-4  Total: 54.17  Rank:9 | Range: 1-3  Total: 48.33  Rank:10 | 48.75 Rank: 13 |
|  |  |  | Fever clearance time | MD -11.11 ( -20.04, -2.18]  ***Favours loading dose*** | OR 2.89  ***Favours loading dose*** | Range: 2-3  Total: 58.33  Rank:8 | Range: 0-3  Total: 54.17  Rank:7 | Range: 0-4  Total: 54.17  Rank:9 | Range: 1-3  Total: 58.33  Rank:7 | 48.75  Rank: 14 |
| 17 | Drugs for treating uncomplicated malaria in pregnant women | Artesunate (AS) vs quinine plus clindamycin (QN+CLD)  (control : QN+CLD) | Treatment failure at 48 hours (excludes new infections, detected by PCR) | Original RR 0.21 (0.12-0.38)  ***Favours AS*** | OR >10  ***Favours AS*** | Range: 1-2  Total: 41.67  Rank:15 | Range: 1-3  Total: 50  Rank:8 | Range: 0-3  Total: 45.83  Rank:11 | Range: 1-3  Total: 50  Rank:9 | 48.44  Rank: 15 |
| 18 | Drugs for treating uncomplicated malaria in pregnant women | Artesunate plus mefloquine (AS+MQ) vs quinine (QN)  (control : QN) | Treatment failure at day 63 (excludes new infections, detected by PCR) | Original RR 0.09 (0.02-0.38) ***favours AS+MQ*** | OR >10  ***favours AS+MQ*** | Range: 0-4  Total: 54.17  Rank:9 | Range: 1-4  Total: 50  Rank:8 | Range: 0-3  Total: 45.83  Rank:11 | Range: 1-3  Total: 45.83  Rank:11 | 46.88  Rank: 16 |
| 19 | Drugs for treating uncomplicated malaria in pregnant women | Sulfadoxine-pyrimethamine (SP) vs chloroquine (CQ)  (control: CQ) | Treatment failure at day 28 | Original RR 0.46 (0.33-0.64)  ***Favours SP*** | OR 2.74 (1.81-4.16)  ***Favours SP*** | Range: 1-4  Total: 70.83  Rank:4 | Range: 1-4  Total: 58.33  Rank:6 | Range: 0-4  Total: 50  Rank:10 | Range:0-3  Total: 45.83  Rank:11 | 46.25  Rank: 17 |
| 20 | Drugs for treating uncomplicated malaria in pregnant women | Artesunate plus atovaquone-proguanil (AS+AP) vs quinine (QN)  (control : QN) | Treatment failure at day 63 | Original RR 0.24 [0.10, 0.57 ] ***favours AS+AP*** | OR 7.87 (2.56-24.17)  ***favours AS+AP*** | Range: 0-3  Total: 54.17  Rank:9 | Range: 1-3  Total: 50  Rank:8 | Range: 0-3  Total: 45.83  Rank:11 | Range: 1-3  Total: 45.83  Rank:11 | 46.25  Rank: 17 |
| 21 | Drugs for treating uncomplicated malaria in pregnant women | Amodiaquine (AQ) vs chloroquine (CQ)  (control : CQ) | Treatment failure at day 28 (excludes new infections, detected by PCR) Study | Original RR 0.20 (0.08-0.46)  ***Favours AQ*** | OR 5.79 (2.35-14.22)  ***Favours AQ*** | Range: 1-4  Total: 66.67  Rank:5 | Range: 1-3  Total: 54.17  Rank:7 | Range: 0-3  Total: 41.67  Rank:12 | Range: 0-3  Total: 50  Rank:9 | 46.25  Rank: 17 |
| 22 | Azithromycin for treating uncomplicated malaria | Azithromycin and chloroquine (AZCQ) vs. atovaquone-proguanil (AP)  (control: atovaquone-proguanil (AP) | Treatment failure day 28, PCR corrected | RR  89.79 [ 5.60, 1440.31 ] ***favours AP*** | OR >10  ***favours AP*** | Range: 2-4  Total: 66.67  Rank:5 | Range: 1-3  Total: 50  Rank:8 | Range: 1-4  Total: 54.17  Rank:9 | Range: 0-4  Total: 45.83  Rank:11 | 43.75  Rank: 18 |
| 23 | Azithromycin for treating uncomplicated malaria | Azithromycin and chloroquine (AZCQ) vs. chloroquine (CQ)  (control: CQ) | Treatment failure day 28, PCR corrected | RR 1.17 [0.09, 14.92]  ***Favours CQ*** | OR 1.2 (0.06-24.47) ***Favours CQ*** | Range: 1-4  Total: 66.67  Rank:5 | Range: 1-3  Total: 50  Rank:8 | Range: 1-4  Total: 45.83  Rank:11 | Range: 0-4  Total: 45.83  Rank:11 | 41.25  Rank: 19 |

**Nutrition**

|  | Review Title | Intervention | Outcome | Original effect size | Effectiveness (Effect Size) | What does this mean? | Feasibility^[[17]](#footnote-17)^ | Deliverability ^[[18]](#footnote-18)^ | Universality ^[[19]](#footnote-19)^ | Effect on Equity^[[20]](#footnote-20)^ | Overall Rating (%) |
| --- | --- | --- | --- | --- | --- | --- | --- | --- | --- | --- | --- |
| 1 | The effects of iodine deficiency in pregnancy and infancy | Iodised oil supplementation | Cretinism | RR 0·27 (0·12–0·60)  ***Favours iodine*** | OR 3.95 (1.70-9.23) ***favours iodine*** | 73% reduced risk of cretinism with salt iodisation | Range: 2-4 Total: 62.5  Rank:3 | Range: 1-4 Total: 50  Rank:6 | Range: 1-4 Total: 58.33  Rank:5 | Range: 2-4 Total: 62.5  Rank:4 | 78.57  Rank: 1 |
| 2 | Kangaroo mother care to reduce morbidity and mortality in low birthweight infants | Kangaroo care vs conventional neonatal care | Mortality at discharge or 40-41 weeks postmenstrual age | RR 0·60, (0·39–0·93)  ***Favours kangaroo care*** | OR 1.72 (1.06-2.79)  ***Favours kangaroo care*** | 40% reduced risk of mortality with kangaroo care for low birthweight babies | Range: 2-4 Total: 62.5  Rank:3 | Range: 2-4 Total: 58.33  Rank:4 | Range: 2-4 Total: 58.33  Rank:5 | Range: 3-4 Total: 66.67  Rank:3 | 73.81  Rank: 2 |
| 3 | Effects and safety of periconceptional folate supplementation for preventing birth defects | Periconceptual folic acid supplementation vs. no treatment/other micronutrient/ placebo | Neural tube defects | RR 0.28 (0.15-0.52)  ***Favours folic acid*** | OR 3.83 (2.10-7.25)  ***Favours folic acid*** | 72% reduced risk of neural tube defects with folic acid | Range: 2-4 Total: 58.33  Rank:4 | Range: 2-4 Total: 54.17  Rank:5 | Range: 3-4 Total: 75  Rank:1 | Range: 2-4 Total: 62.5  Rank:4 | 73.81  Rank: 3 |
| 4 | Daily oral iron supplementation during pregnancy | Any supplements containing iron versus same supplements without iron or no treatment/placebo | Low Birthweight | RR 0.81 (0.68-0.97)  ***Favours iron*** | OR 1.24 (1.07-1.43)  ***Favours iron*** | 19% reduced risk of low birthweight with iron-folate | Range: 3-4 Total: 75  Rank:1 | Range: 2-4 Total: 70.83  Rank:1 | Range: 3-4 Total: 70.83  Rank:2 | Range: 3-4 Total: 75  Rank:1 | 73.81  Rank: 4 |
| 5 | Calcium supplementation during pregnancy for preventing hypertensive disorders and related problems | Routine high-dose calcium supplementation in pregnancy | Pre-eclampsia | RR 0.45 (0.31-0.65)  ***favours calcium*** | OR 1.36 (1.19-1.56)  ***favours calcium*** | 55% reduced risk of pre-eclampsia for mothers who received | Range: 1-4 Total: 58.33  Rank:4 | Range: 1-4 Total: 58.33  Rank:4 | Range: 2-4 Total: 66.67  Rank:3 | Range: 2-4 Total: 66.67  Rank:3 | 71.43  Rank: 5 |
| 6 | Oral zinc for treating diarrhoea in children | Zinc supplementation | Diarrhea on day 5 | RR 0.67 (0.51-0.89)  ***Favours zinc*** | OR 1.57 (1.15-2.15)  ***Favours zinc*** | 33% reduced risk of diarrhea on day 5 when zinc given to children | Range: 1-4 Total: 58.33  Rank:4 | Range: 2-4 Total: 54.17  Rank:5 | Range: 2-4 Total: 62.5  Rank:4 | Range: 2-4 Total: 70.83  Rank:2 | 69.05  Rank: 6 |
| 7 | Vitamin A supplementation for preventing morbidity and mortality in children from 6 months to 5 years of age | Vitamin A supplementation | All cause mortality Follow up : 12-96 weeks | RR  0.76 (0.69 to 0.83)  ***Favours vit a*** | OR 1.42 (1.15-1.75)  ***Favours vit a*** | 24% reduced risk of mortality with vitamin A supplementation. | Range: 3-4 Total: 70.83  Rank:2 | Range: 3 Total: 62.5  Rank:3 | Range: 3-4 Total: 66.67  Rank:3 | Range: 3-4 Total: 70.83  Rank:2 | 66.67  Rank: 7 |
|  |  | Vitamin A supplementation | Diarrhea-related mortality | RR 0.72 (0.57-0.91)  ***Favours vit a*** | OR 1.92 (1.52- 2.78)  ***Favours vit a*** | 28% reduced risk of diarrhea-related mortality with vitamin A supplementation |  |  |  |  |  |
| 8 | Vitamin A for treating measles in children | Vitamin A vs placebo | Mortality - age 2 years or less | Original RR 0.21 (0.07-0.66)  ***Favours vitamin a*** | OR 5.95 (1.71-20.74)  ***Favours vitamin a*** | 79% reduced risk of mortality with vitamin A in children with measles. | Range: 2-4  Total: 75  Rank:1 | Range: 2-3  Total: 66.67  Rank:2 | Range: 2-4  Total:54.17  Rank:6 | Range: 2-4  Total: 75  Rank:1 | 61.91  Rank: 8 |
| 9 | Zinc supplementation for the prevention of pneumonia in children aged 2 months to 59 months | Zinc supplementation | Overall pneumonia incidence reduction | RR 0.87 (0.81-0.94)  ***Favours zinc*** | OR 1.65 (1.33-2.05)  ***Favours zinc*** | 13% reduced pneumonia incidence when zinc supplementation given to children between 2 and 59 months. | Range: 2-4 Total: 50  Rank:6 | Range: 2-4 Total: 54.17  Rank:5 | Range: 2-4 Total: 54.17  Rank:6 | Range: 2-4 Total: 66.67  Rank:3 | 61.90  Rank: 9 |
| 10 | Impact of maternal education about complementary feeding and provision of complementary foods on child growth in developing countries. | Maternal education +provision of complementary foods | Stunting | OR 0.71 (0.56-0.92)  ***Favours education + complementary foods*** | OR 1.41 (1.09-1.79)  ***Favours education + complementary foods*** | 29% reduced risk of stunting when maternal education +complementary foods provided. | Range: 1-4 Total: 54.17  Rank:5 | Range: 1-4 Total: 45.83  Rank:7 | Range: 3-4 Total: 62.5  Rank:4 | Range: 2-4 Total: 62.5  Rank:4 | 61.90  Rank: 10 |

Table 2: Results for all criteria

|  |  | **Feasibility (score out of 100)** | **Deliverability (score out of 100)** | **Universality (out of 100)** | **Effect on Equity (score out of 100)** | **Overall Rating (score out of 100)** |
| --- | --- | --- | --- | --- | --- | --- |
| **Depression** | Highest Score | 59.38  Tricyclic antidepressants (TCAs) vs. placebo for depression symptoms post-treatment  Rank: 1^st^ | 59.38  TCAs vs. placebo for depression symptoms post-treatment  Rank: 1st | 65.63  Relaxation vs. wait-list/no treatment/minimal treatment  Rank: 10^th^ | 65.63  Psychosocial and psychological interventions for treating postpartum depression vs. usual care  Rank: 3^rd^ | 86  TCAs vs. placebo for depression in primary care  Rank: 1^st^ for feasibility and deliverability, 3^rd^ for universality, and 2^nd^ for effect on equity |
|  | Lowest Score | 31.25  Psychological/educational intervention versus no intervention/wait-list/usual care for depression score  Rank: 6^th^  Milnacipran vs TCAs for depression for response at acute phase (6-12 weeks)  Rank: 21^st^ | 31.25 Psychological/educational interventions vs. no intervention/wait list/usual care for preventing depression in children and adolescents Rank: 6^th^  Milnacipran vs. TCAs for response at acute phase Rank: 21^st^  Alprazolam vs. placebo for 50% improvement vs. less than 50% improvement  Rank: 22^nd^ | 31.25  Milnacipran vs. TCAs for response at acute phase  Rank: 21^st^  Alprazolam vs. placebo for 50% improvement Rank: 22nd  Alprazolam vs. amitriptyline for 50% improvement  Rank: 22^nd^ | 28.13  Escitalopram vs. citalopram for reduction of depressive symptoms  Rank: 20^th^  Milnacipran vs. TCAs Rank: 21^st^  Alprazolam vs. placebo Rank: 22^nd^ | 19.23  Alprazolam vs. amitriptyline  Rank: 8^th^ for feasibility, 10^th^ for deliverability, 13^th^ for universality and eighth for effect on equity |
| **Diabetes/obesity** | Highest Score | 87.5  Suphonylureas versus insulin for patients with type 2 diabetes mellitus Rank: 2^nd^ | 87.5  Metformin vs. sulphonylureas or insulin Rank: 2nd | 85  ACEi vs. placebo/no treatment for preventing diabetic kidney disease Rank: 3^rd^ | 81.25  Group-based diabetes education program vs. individual routine treatment for reduction in diabetes medication  Rank: 5^th^ | 76.67  Sulphonylureas vs. insulin for patients with type 2 diabetes mellitus  Rank: 3^rd^ for feasibility, 1^st^ for deliverability, 5^th^ for universality, and 7^th^ for effect on equity |
|  | Lowest Score | 37.5  Complex interventions for preventing diabetic foot ulceration  Rank: 26^th^ | 40  Insulin once daily vs. insulin plus oral antihyperglycaemic agents for HbA1c change from baseline  Rank: 29^th^ | 50  Insulin once daily vs. insulin once daily plus oral antihyperglycaemic agents for HbA1c change from baseline  Rank: 29^th^ | 50  Insulin once daily vs. insulin once daily plus oral hyperglycaemic agents for patients with type 2 diabetes mellitus  Rank: 29^th^ | 23.81  Scaling, root planing and oral hygiene vs. no/usual treatment for glycaemic control in people with diabetes  Rank: 11^th^ for feasibility, 19^th^ for deliverability, 10^th^ for universality, and 6^th^ for effect on equity |
| **HIV/AIDS** | Highest Score | 87.5  Prevention of diarrhea in children with HIV infection (or exposure) using vitamin A vs. placebo for mortality  Rank 1^st^  Cotrimoxazole prophylaxis for opportunistic infections Rank 3^rd^  Deworming of co-infected individuals for delaying HIV disease progression (change in log10 HIV-1 RNA)  Rank 6^th^ | 87.5  Four interventions: prevention of diarrhea in children with vitamin A vs. placebo for mortality (all cause)  Rank: 1^st^  Antihelminthic treatment for deworming co-infected individuals on chance in log10 HIV-1 RNA  Rank: 6^th^  Tenofovir (TDF) vs. zidovudine (AZT) for virologic and immunological response Rank: 17^th^  Mass media interventions – leaflets vs. controls for update of HIV testing Rank: 19^th^ | 95.83  Cotrimoxazole prophylaxis for opportunistic infections vs. control  Rank: 3^rd^ | 87.5  Vitamin A vs. placebo for prevention of diarrhea in children with HIV infection of exposure to maternal HIV infection  Rank: 1^st^  Promotion of female and male condoms vs. promotion of male condoms  Rank: 2^nd^  AZT lamivudine and lopinavir/ritonavir starting at 28-36 weeks gestation vs. short course regimen  Rank: 5^th^ | 96.25  Vitamin A vs. placebo for prevention of diarrhea in children with HIV infection or exposure to maternal HIV infection  Rank: 1^st^ for feasibility, deliverability, effect on equity, and 2^nd^ for universality |
|  | Lowest Score | 37.5  Male circumcision for prevention of homosexual acquisition of HIV  Rank: 15^th^ | 40  AZT plus zalcitabine vs. AZT alone  Rank: 39^th^ | 29.17  Highly active antiretroviral therapy (HAART) plus doxorubicin, bleomycin and vincristine (ABV) vs. HAART alone for treatment of severe or progressive Kaposi’s sarcoma  Rank: 29^th^ | 35  AZT plus zalcitabine vs. AZT alone in HIV-infected adults  Rank: 39^th^ | 22.22  AZT plus zalcitabine vs. AZT alone in HIV-infected adults  Rank: 9^th^ for feasibility, 14^th^ for deliverability, 15^th^ for universality, and 18^th^ for effect on equity |
| **Malaria** | Highest Score | 87.5  Tablets in sectioned polythene bags vs. bottled syrup for treatment adherence Rank 1^st^ | 79.17  Tablets in sectioned polythene bags vs. bottled syrup for treatment adherence Rank: 1^st^ | 83.33  Artemether-lumefantrine (AL) vs. amodiaquine plus sulfadoxine-pyrimethamine for treating uncomplicated malaria  Rank: 2nd | 95.83  Home- or community-based programs for treating malaria vs. standard care  Rank: 3^rd^ | 84.06  Tablets in sectioned polythene bags vs. bottled syrup for treating malaria  Rank: 1^st^ for feasibility, deliverability, 8^th^ for universality, and 2^nd^ for effect on equity |
|  | Lowest Score | 41.67  Artesunate vs. quinine plus clindamycin got uncomplicated malaria in pregnant women  Rank: 15^th^ | 50  Intermittent preventive treatment of malaria for children in areas with high seasonal transmission for severe malaria  Rank: 5^th^  Amodiaquine plus sulfadoxine-pyrimethamine vs. chloroquine for treating uncomplicated malaria in pregnant women  Rank: 12^th^  Artesunate vs. quinine plus clindamycin for treating uncomplicated malaria in pregnant women  Rank: 15^th^  Artesunate plus mefloquine vs. quinine for treating uncomplicated malaria in pregnant women  Rank: 16^th^  Artesunate plus atovaquone-proguanil vs. quinine for treating uncomplicated malaria in pregnant women  Rank: 17^th^  Azithromycin and chloroquine vs. atovaquone-proguanil for treating uncomplicated malaria  Rank: 18^th^  Azithromycin and chloroquine vs. chloroquine for treating uncomplicated malaria Rank: 19^th^ | 37.5  Amodiaquine plus sulfadoxine-pyrimethamine vs. chloroquine for treating uncomplicated malaria in pregnant women  Rank: 12^th^ | 45.83  Artesunate plus mefloquine vs. quinine for treating uncomplicated malaria in pregnant women  Rank: 16^th^  Sulfadoxine-pyrimethamine vs. chloroquine for treating uncomplicated malaria in pregnant women  Rank: 17^th^  Artesunate plus atovaquone-proguanil vs. quinine for treating uncomplicated malaria in pregnant women  Rank: 17^th^  Azithromycin and chloroquine vs. atovaquone-proguanil for treating uncomplicated malaria in pregnant women  Rank: 18^th^  Azithromycin and chloroquine vs. chloroquine treating uncomplicated malaria  Rank: 19^th^ | 41.25  Azithromycin and chloroquine vs. chloroquine for treating uncomplicated malaria  Rank: 5^th^ for feasibility, 8^th^ for deliverability, and 11^th^ for universality and effect on equity |
| **Nutrition** | Highest Score | 75  Daily oral iron supplementation during pregnancy vs the same supplement without iron/no treatment/placebo for low birthweight  Rank: 4^th^ | 70.83  Supplements containing iron vs. supplements without iron/no treatment/placebo on low birthweight  Rank: 4^th^ | 75  Periconceptual folic acid supplementation vs. no other treatment/other micronutrient/placebo for preventing neural tube defects  Rank: 3^rd^ | 75  Daily supplement containing iron vs. supplements without iron/no treatment/placebo  Rank: 4^th^  Vitamin A vs. placebo for treating measles in children  Rank: 8^th^ | 78.57  Iodised oil supplementation in pregnancy and infancy  Rank: 3^rd^ for feasibility, 6^th^ for deliverability, 5^th^ for universality, and fourth for effect on equity |
|  | Lowest Score | 50 Zinc supplementation for overall pneumonia incidence reduction  Rank: 9^th^ | 45.83  Maternal education plus provision of complementary goods for stunting  Rank: 10^th^ | 54.17  Both vitamin A vs. placebo for treating measles in children  Rank: 8^th^  Zinc supplementation for the prevention of pneumonia in children aged 2 to 59 months Rank: 9^th^ | 62.5  Maternal education plus provision of complementary foods on stunting Rank: 10^th^ | 61.90  Maternal education plus provision of complementary foods for child growth in developing countries  Rank: 5^th^ for feasibility, 7^th^ for deliverability, and 4^th^ for universality and effect on equity |

1. Is there sufficient capacity to implement the intervention? Is it feasible to provide required training to staff? Rankings are 0 to 4. 4= optimal (easier to implement), 0=more difficult [↑](#footnote-ref-1)
2. Consider the level of difficulty with intervention delivery, the infrastructure required (human resources, facilities, etc.). Consider the resources available and whether the intervention is affordable. Rank 0-4, 4= optimal (easier/fewer health system effects), 0=more difficult/greater health system effects, [↑](#footnote-ref-2)
3. Is the intervention relevant to most countries? Rankings are 0 to 4. 4= Optimal (more generalizable/population-based, 0= less generalizable/specific population [↑](#footnote-ref-3)
4. Does the distribution of the disease burden affect mainly the disadvantaged? Are the disadvantaged most likely to benefit from the intervention? Will the intervention improve equity in disease burden distribution long-term? Rankings are 0 to 4. 4= Optimal (more generalizable/population-based, 0= less generalizable/specific population [↑](#footnote-ref-4)
5. Is there sufficient capacity to implement the intervention? Is it feasible to provide required training to staff? Rankings are 0 to 4. 4= optimal (easier to implement), 0=more difficult [↑](#footnote-ref-5)
6. Consider the level of difficulty with intervention delivery, the infrastructure required (human resources, facilities, etc.). Consider the resources available and whether the intervention is affordable. Rank 0-4, 4= optimal (easier/fewer health system effects), 0=more difficult/greater health system effects, [↑](#footnote-ref-6)
7. Is the intervention relevant to most countries? Rankings are 0 to 4. 4= Optimal (more generalizable/population-based, 0= less generalizable/specific population [↑](#footnote-ref-7)
8. Does the distribution of the disease burden affect mainly the disadvantaged? Are the disadvantaged most likely to benefit from the intervention? Will the intervention improve equity in disease burden distribution long-term? Rankings are 0 to 4. 4= Optimal (more generalizable/population-based, 0= less generalizable/specific population [↑](#footnote-ref-8)
9. Is there sufficient capacity to implement the intervention? Is it feasible to provide required training to staff? Rankings are 0 to 4. 4= optimal (easier to implement), 0=more difficult [↑](#footnote-ref-9)
10. Consider the level of difficulty with intervention delivery, the infrastructure required (human resources, facilities, etc.). Consider the resources available and whether the intervention is affordable. Rank 0-4, 4= optimal (easier/fewer health system effects), 0=more difficult/greater health system effects, [↑](#footnote-ref-10)
11. Is the intervention relevant to most countries? Rankings are 0 to 4. 4= Optimal (more generalizable/population-based, 0= less generalizable/specific population [↑](#footnote-ref-11)
12. Does the distribution of the disease burden affect mainly the disadvantaged? Are the disadvantaged most likely to benefit from the intervention? Will the intervention improve equity in disease burden distribution long-term? Rankings are 0 to 4. 4= Optimal (more generalizable/population-based, 0= less generalizable/specific population [↑](#footnote-ref-12)
13. Is there sufficient capacity to implement the intervention? Is it feasible to provide required training to staff? Rankings are 0 to 4. 4= optimal (easier to implement), 0=more difficult [↑](#footnote-ref-13)
14. Consider the level of difficulty with intervention delivery, the infrastructure required (human resources, facilities, etc.). Consider the resources available and whether the intervention is affordable. Rank 0-4, 4= optimal (easier/fewer health system effects), 0=more difficult/greater health system effects, [↑](#footnote-ref-14)
15. Is the intervention relevant to most countries? Rankings are 0 to 4. 4= Optimal (more generalizable/population-based, 0= less generalizable/specific population [↑](#footnote-ref-15)
16. Does the distribution of the disease burden affect mainly the disadvantaged? Are the disadvantaged most likely to benefit from the intervention? Will the intervention improve equity in disease burden distribution long-term? Rankings are 0 to 4. 4= Optimal (more generalizable/population-based, 0= less generalizable/specific population [↑](#footnote-ref-16)
17. Is there sufficient capacity to implement the intervention? Is it feasible to provide required training to staff? Rankings are 0 to 4. 4= optimal (easier to implement), 0=more difficult [↑](#footnote-ref-17)
18. Consider the level of difficulty with intervention delivery, the infrastructure required (human resources, facilities, etc.). Consider the resources available and whether the intervention is affordable. Rank 0-4, 4= optimal (easier/fewer health system effects), 0=more difficult/greater health system effects, [↑](#footnote-ref-18)
19. Is the intervention relevant to most countries? Rankings are 0 to 4. 4= Optimal (more generalizable/population-based, 0= less generalizable/specific population [↑](#footnote-ref-19)
20. Does the distribution of the disease burden affect mainly the disadvantaged? Are the disadvantaged most likely to benefit from the intervention? Will the intervention improve equity in disease burden distribution long-term? Rankings are 0 to 4. 4= Optimal (more generalizable/population-based, 0= less generalizable/specific population [↑](#footnote-ref-20)
