# Supplementary figures and images for: Setting priorities for knowledge translation of Cochrane reviews for health equity: Evidence for Equity
Source: Int J Equity Health. 2017 Dec 2;16:208. doi: 10.1186/s12939-017-0697-5 (PMC5712153; doi:10.1186/s12939-017-0697-5)

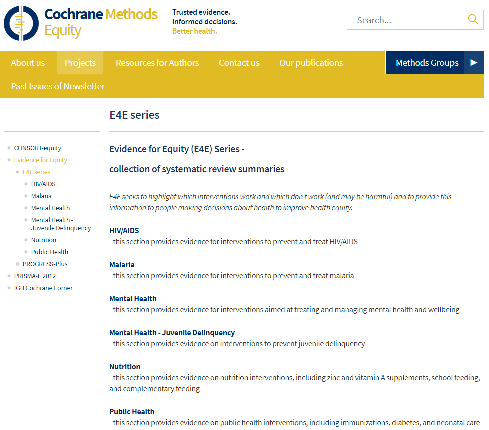

Supplement: Supplementary file 4 — Screenshot of E4E Landing Page and. (PNG 23 kb) [file 12939_2017_697_MOESM4_ESM.png]

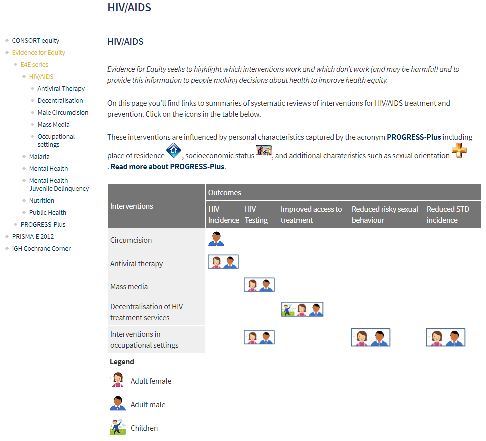

Supplement: Supplementary file 5 — Screenshot of HIV Topic Landing Page. (PNG 32 kb) [file 12939_2017_697_MOESM5_ESM.png]
